# Conductive Bacterial Nanocellulose-Polypyrrole Patches Promote Cardiomyocyte Differentiation

**DOI:** 10.1021/acsabm.3c00303

**Published:** 2023-06-21

**Authors:** Sumithra Yasaswini Srinivasan, Marina Cler, Osnat Zapata-Arteaga, Bernhard Dörling, Mariano Campoy-Quiles, Elena Martínez, Elisabeth Engel, Soledad Pérez-Amodio, Anna Laromaine

**Affiliations:** † Institute of Material Science of Barcelona (ICMAB), CSIC, Campus UAB, 08193 Bellaterra, Spain; ‡ IMEM-BRT Group, Departament de Ciència i Enginyeria de Materials, Universitat Politecnica de Catalunya, 08028 Barcelona, Spain; § Biomimetic Systems for Cell Engineering, Institute for Bioengineering of Catalonia (IBEC), The Barcelona Institute of Science and Technology, 08028 Barcelona, Spain; ⊥ CIBER en Bioingenieria, Biomateriales y Nanomedicina, CIBER-BBN, 28029 Madrid, Spain; ¶ Biomaterials for Regenerative Therapies, Institute of Bioengineering Catalunya (IBEC), The Barcelona Institute of Science and Technology, 08028 Barcelona, Spain; # Department of Electronics and Biomedical Engineering, University of Barcelona (UB), 08028 Barcelona, Spain

**Keywords:** bacterial nanocellulose, polypyrrole, conducting polymers, tissue engineering, cardiac patches

## Abstract

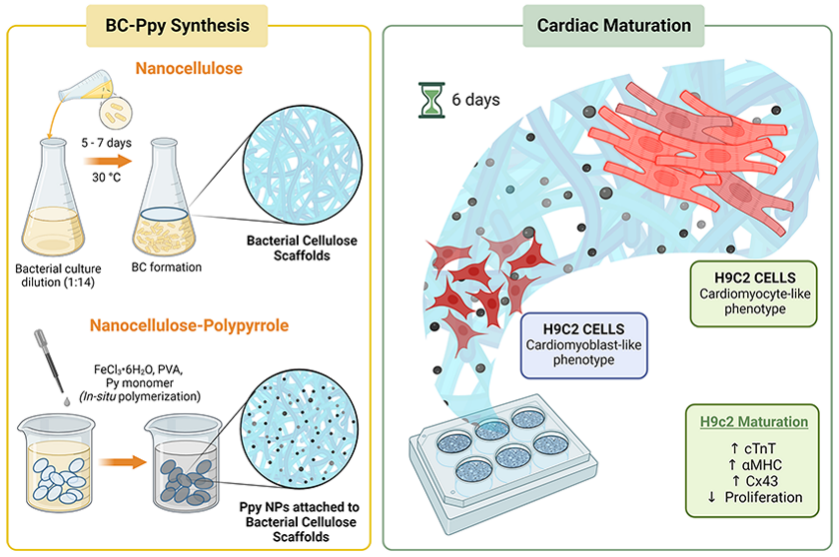

The low endogenous regenerative capacity of the heart, added to the prevalence of cardiovascular diseases, triggered the advent of cardiac tissue engineering in the last decades. The myocardial niche plays a critical role in directing the function and fate of cardiomyocytes; therefore, engineering a biomimetic scaffold holds excellent promise. We produced an electroconductive cardiac patch of bacterial nanocellulose (BC) with polypyrrole nanoparticles (Ppy NPs) to mimic the natural myocardial microenvironment. BC offers a 3D interconnected fiber structure with high flexibility, which is ideal for hosting Ppy nanoparticles. BC-Ppy composites were produced by decorating the network of BC fibers (65 ± 12 nm) with conductive Ppy nanoparticles (83 ± 8 nm). Ppy NPs effectively augment the conductivity, surface roughness, and thickness of BC composites despite reducing scaffolds’ transparency. BC-Ppy composites were flexible (up to 10 mM Ppy), maintained their intricate 3D extracellular matrix-like mesh structure in all Ppy concentrations tested, and displayed electrical conductivities in the range of native cardiac tissue. Furthermore, these materials exhibit tensile strength, surface roughness, and wettability values appropriate for their final use as cardiac patches. *In vitro* experiments with cardiac fibroblasts and H9c2 cells confirmed the exceptional biocompatibility of BC-Ppy composites. BC-Ppy scaffolds improved cell viability and attachment, promoting a desirable cardiomyoblast morphology. Biochemical analyses revealed that H9c2 cells showed different cardiomyocyte phenotypes and distinct levels of maturity depending on the amount of Ppy in the substrate used. Specifically, the employment of BC-Ppy composites drives partial H9c2 differentiation toward a cardiomyocyte-like phenotype. The scaffolds increase the expression of functional cardiac markers in H9c2 cells, indicative of a higher differentiation efficiency, which is not observed with plain BC. Our results highlight the remarkable potential use of BC-Ppy scaffolds as a cardiac patch in tissue regenerative therapies.

## Introduction

Cardiovascular diseases (CVD) are the leading cause of death globally, accounting for nearly 45% of all deaths in Europe.^1,2^ Cardiac arrhythmia (CA) is a prevalent condition occurring in as much as 90% of primary heart patients.^3^ One of the primary causes of CA is myocardial infarction (MI), where a region of the cardiac muscle is damaged.^4^ The heart has a limited regenerative ability;^5^ consequently, cardiomyocytes lost due to MI cannot be replaced. Heart transplantation is currently the best available long-lasting treatment for severely damaged hearts. However, the lack of donor organs, compatibility requirements, the need for life-long immune suppression, and host immune rejection are still serious hurdles,^6,7^ which have promoted the rise of cardiac tissue engineering (TE).

To regenerate damaged myocardial tissue, TE employs cells, scaffolds, and growth factors (or a combination of these).^8^ Researchers have investigated the possibility of injecting viable cells, such as stem or progenitor cells, into the infarcted myocardium to induce myocyte regeneration.^9,10^ However, this approach is limited by poor cell viability, low cell retention at the affected area, cell aggregation, and the inability to generate new functional cardiac tissues.^11−14^ The employment of scaffolds that can serve as delivery platforms has improved cell behavior *in vivo* and *in vitro* and enhanced the therapeutic effect of cell-based therapies.^10,15^ Inferred outcomes are directly dependent on the intrinsic properties of the engineered biomaterials; therefore, a proper design considering the requirements for its final application to the native tissue is crucial.

Topographical, biochemical, and electrical cues at the micro- and nanoscale are essential determinants of cardiac organization, morphology, and electrical and mechanical function. The heart is an electrically conducting organ, mediated by ionic diffusion at the neuromuscular junction and within the cardiomyocytes that generate action potentials and propagate electrical signals, leading to muscle contraction. Therefore, a scaffold for cardiac TE should mimic the native cardiac microenvironment, be cytocompatible, and possess appropriate mechanical and electrical properties to promote cell organization, survival, and function in the damaged host cardiac tissue.^16,17^ Chitosan, collagen, alginate, and extracellular matrix (ECM) from decellularized cardiac tissue are biomaterials explored in cardiac TE.^15−19^ However, none of these materials possesses electrical conductivity and thus they do not adequately mimic the intrinsic characteristics of the natural myocardial microenvironment. To tackle this, current approaches employing engineered cardiac scaffolds with electroconductive biomaterials, such as intrinsically conducting conjugated polymers (CPs), are being actively researched.^16,20−26^

Among CPs, polypyrrole (Ppy) is a popular candidate since it can be synthesized using a simple oxidative polymerization method, is dispersible in water with good aqueous stability, is biocompatible, possesses tunable electrical conductivity, and can be combined with a large variety of biomaterials to form bionanocomposites.^22,27−29^ However, like most other CPs, PPy is insoluble and mechanically rigid on its own, which can affect its interaction with the elastic tissues of the heart. This work proposes a strategy combining Ppy with bacterial cellulose to produce cytocompatible, conductive, and flexible scaffolds for cardiac TE. Bacterial cellulose (BC) is a natural polymer with high purity compared to plant-derived cellulose, high porosity, and water retention capacity. BC is biocompatible,^30^ produced with low endotoxin levels (0.04 ± 0.01 endotoxin units (EU)/mL),^31^ nonimmunogenic,^32−34^ and not biodegradable, thus offering longer residence time on the heart surface compared to natural hydrogel systems. BC films are highly flexible with excellent mechanical strength (up to 250 MPa) to withstand chronic shear stress and repeated contractile force. The fibrous mesh-like microstructure of BC highly resembles the extracellular matrix,^30^ mimicking the natural myocardial microenvironment, and incorporating Ppy in the BC films imparts electrical properties.^35−38^ Scaffolds combining plant-derived cellulose and Ppy have been reported for neuronal tissue engineering^37,39^ and cardiac applications;^40,41^ however, the use of bacterial cellulose, BC-Ppy, has not been evaluated for cardiac TE purposes.

BC-Ppy composites produced in this work are assessed using primary cardiac fibroblasts and H9c2 cells. We have highlighted the ability of BC-Ppy scaffolds to promote H9c2 differentiation to evaluate the materials in realistic environments resembling the adult cardiac tissue, which we believe is cardinal to ensure a high translational value of the cardiac patches.

## Materials and Methods

### BC Films

A commercial *Komagataeibacter xylinus* strain (NCIMB 5346, CECT, Valencia, Spain) was used for BC production. The bacterial strain was maintained in agar plates with Hestrin-Schramm (HS) medium as the growth media containing peptone, agar, yeast, glucose (Condalab), citric acid, and Na_2_HPO_4_·12H_2_O (Sigma-Aldrich). Bacterial solutions were diluted with fresh media (1:14 ratio) and were maintained for 5–7 days in culture well plates, as previously described.^42^ BC films were carefully harvested and washed once in 50% ethanol (Panreac), twice in boiling 0.1 M NaOH (Sigma-Aldrich), and then rinsed with water to obtain neutral pH. BC films were stored at room temperature in water until use. For material characterization, BC films were dried at 60 °C for 2–3 days by placing them between Teflon plates under a 1 kg weight.

### BC-Ppy Films

Ppy nanoparticles (NPs) were synthesized using *in situ* oxidative polymerization in the presence of presynthesized BC films, as previously described.^43^ For the BC-Ppy synthesis, pyrrole (Sigma-Aldrich), poly(vinyl alcohol) (PVA) (average Mw: 30,000–70,000, 87–90% hydrolyzed, Sigma-Aldrich), and FeCl_3_·6H_2_O (Merck) were used. Wet BC films were added to an aqueous solution containing 7.5% PVA (wt/wt of monomer) as the surfactant and ferric chloride hexahydrate as the oxidant (2.4 times Py monomer concentration) and stirred slowly for 1 h to allow absorption of the reactants into the BC films. Then pyrrole monomer (1–100 mM) was added dropwise to it under slow stirring, and the polymerization was carried out for 4 h at room temperature. The resulting BC-Ppy films were washed in distilled water until the supernatant was clear. The BC-Ppy films were dried at 60 °C for 2–3 days by placing them in between Teflon plates with a 1 kg weight on top. *In vitro* experiments were performed in dried BC composites to facilitate sterilization procedures before cell culture. Nevertheless, some cell culture experiments were conducted as well with BC composites prior to the drying process (referred to as “wet” materials). This was done to confirm akin behavior of the composites before and after drying treatment.

### Scanning Electron Microscopy (SEM)

BC-Ppy films were blended for 30 min and centrifuged at 9000 rpm for 15 min. The pellet was then dried at 60 °C and crushed using a pestle and mortar to yield powder. The powder was dispersed in water and sonicated for 5–10 min before sample preparation. The liquid was dropped on carbon tape mounted over an aluminum sample holder for SEM characterization and imaged in a scanning electron microscope (QUANTA FEI 200 FEG-ESEM, voltage of 20 kV, working distance of 8 mm, magnification levels of 10, 20, 40, and 100 kX).

### Transmission Electron Microscopy (TEM)

TEM samples were prepared in the same manner as SEM samples. A single drop of BC-Ppy aqueous dispersion was placed on top of a copper grid and dried at room temperature prior to imaging. Samples were imaged with a transmission electron microscope (JEOL JEM-1210, voltage of 120 kV, magnification levels of 3, 6, 8, 10, and 20 kX). The sizes of BC fibers and Ppy NPs from TEM images were analyzed using ImageJ software.

### Surface Roughness

The effect of Ppy on the surface roughness of the composites was studied using atomic force microscopy (AFM). BC and BC-Ppy (1 mM) composites were prepared as usual and dried directly on top of a cover glass at room temperature. The films adhered strongly to the glass cover enabling scanning by the AFM probe on the surface. The scanning was performed in tapping mode, for a 30 × 30 μm^2^ area, and at least 3 regions per sample and 3 samples per concentration were scanned.

### FTIR and UV–vis/NIR Spectroscopy

The chemical structure and composition of the as-synthesized Ppy and BC-Ppy were characterized by attenuated total reflectance-Fourier transform infrared (ATR-FTIR) spectroscopy (Spectrophotometer Jasco 4700) at a spectral range of 400–4000 cm^–1^. The absorbance spectra of Ppy NPs and BC-Ppy were measured using a Cary 5000 UV–vis/NIR spectrophotometer at a wavelength range of 200–1600 nm. Ppy NPs were dispersed in water by sonication and analyzed in liquid form, whereas BC-Ppy films were prepared at a 5 mM concentration to yield semitranslucent BC-Ppy films to allow light transmission.

### Electrical Conductivity

The sheet resistance of dried BC-Ppy films was measured using an in-house 4-probe resistance measuring system using the Van der Pauw method.^44^ Film thickness was measured using a digital micrometer by choosing 5 points of contact on each film and taking the average. The electrical conductivity σ can then be calculated as follows:
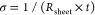
where *R*_sheet_ is the sheet resistance of each film and *t* is its average thickness. The electrical conductivity of high-concentration materials (10, 50, and 100 mM) was measured as individual films. However, lower concentrations possessed conductivities below the range of our instruments. Consequently, at lower concentrations (1, 3, and 5 mM), five BC-Ppy films were stacked and dried together, forming a single thicker film with conductivity in the detectable range. Then we computed the conductivity of the stacked films and extrapolated it to obtain the values of the individual films.

### Stability of BC-Ppy

The stability of BC-Ppy materials was evaluated by visual observation, UV–vis/NIR, and conductivity at different conditions. 0.1 M BC-Ppy nanocomposites, which had the highest concentration of Ppy tested, were employed for this analysis. First, the materials were evaluated over time: BC-Ppy was stored for six months at room temperature in sterile milli-Q water. The supernatant was collected, and UV–vis/NIR scan was performed to analyze whether any Ppy-NPs had leached out from the composites into the supernatant. Further, the leaching of Ppy NPs into the supernatant was also assessed after 30 min of UV sterilization on each side of the films and after autoclaving at 121 °C for 20 min. The stability was also evaluated after incubation in the cell culture medium. BC-Ppy materials were incubated in Dulbecco’s modified Eagle’s medium (DMEM, Gibco, Invitrogen) medium for 24 h at 37 °C. Then culture medium was collected, and the presence of Ppy NPs in the supernatant was investigated. BC-Ppy scaffolds were then dried for 24 h after being subjected to these four processes (water storage, UV sterilization, autoclaving, and immersion in cell culture media). Following this, conductivity was measured and compared to the initial samples.

### Mechanical Properties

The tensile strength and breaking strain of the materials were measured by cyclic uniaxial tensile and biaxial tensile tests. For both, Instron MicroTester 5548 (University of Zaragoza) at a test speed of 5 mm/min was used with a 5N and 10 N load cell for the uniaxial and biaxial tests, respectively. For uniaxial tests, BC and BC-Ppy at different concentrations were grown to dog-bone shapes using 3D-printed molds and were clamped at the two ends. Under submerged conditions, the material was pulled from both ends with a 60 mm distance between the clamps until the samples experienced a fracture and failed (Figure S1A). We measured the increase in the sample’s length after elongation. Here, the measured length was the distance between two holding clamps and not the total length of the sample. Load vs. deformation data was obtained and then converted to stress vs strain according to the formulas:
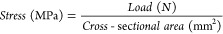

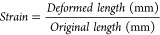


From the stress–strain curve, the maximum stress that a sample can withstand before failure is defined as its tensile strength. The strain at which sample fracture occurs is referred to as breaking strain, and the initial slope of the curve represents Young’s modulus of a material. These properties together define the stiffness of a given material.

For the biaxial tensile test, the material was pulled at a uniform rate from all four directions. Therefore, two load-deformation curves were obtained from the two axes. The load-deformation was converted to stress vs. strain using the above formula, where the initial distance between the clamps was 9.5 mm (Figure S1B).

### Surface Wettability

The surface wettability was assessed through contact angle measurements for BC, and BP1, BP3, BP5, and BP10 composite materials. The films were dried and rewet in Milli-Q water for 24 h. The rehydrated films were placed on top of a glass slide to facilitate adhesion of the films on the glass and provide a flat surface for a precise angle measurement. The adhered films were placed on the platform and a drop (4 μL) of either DMEM or water was deposited at a rate of 1185 μL/min on top to measure the DMEM/water contact angle, respectively. The solvent drop on the surface was captured and angles were measured using a Drop Shape Analyzer DSA 100 (KRÜSS).

### Cell Culture

Low-adherent well plates were prepared by coating them with 5 mg/mL poly(2-hydroxyethyl methacrylate) solution (Sigma) and stored sealed at 4 °C until use. BC and BC-Ppy materials were sterilized with UV irradiation for 15 min on each side and secured by polytetrafluoroethylene rings at the bottom of each well. The scaffolds were immersed in DMEM containing 4.5 g/L glucose, 50% FBS (Gibco, Invitrogen), and incubated overnight inside a cell culture incubator at 37 °C with 5% CO_2_. Scaffolds were coated with collagen (100 mg/mL, VitroCol, Advanced Biomatrix) prior to myoblast seeding.

Thirty thousand cardiomyoblasts/cm^2^ or fifteen thousand cardiac fibroblasts/cm^2^ were seeded on top of each scaffold. Culture plastic wells (without scaffolds) were used as controls. Adult human cardiac fibroblasts (p5-p6, PromoCell) were cultured in fibroblast growth medium (PromoCell), whereas H9c2 rat cardiac myoblasts (p2-p9, ATCC) were cultured with DMEM containing 4.5 g/L glucose, 10% FBS, 1% l-glutamine (Gibco, Thermo Fisher), and 1% penicillin/streptomycin (Gibco, Invitrogen). Cultures were maintained in a humidified atmosphere with 5% CO_2_ at 37 °C, and culture medium was changed every 2 days.

### Cell Viability

The cytocompatibility of BC composites was examined through a LIVE/DEAD Assay. After the cells were cultured onto the materials, these were washed in DPBS (Gibco, Thermo Fisher) and incubated in a solution of 2 μM calcein-AM (Sigma) and 4 μM propidium iodide (Fluka) in DPBS for 20 min at 37 °C. Cells were then washed in DPBS and immediately imaged using a motorized wide-field microscope Leica Thunder 3D Live Cell. The number of alive and dead cells was quantified using ImageJ software. For analysis, a minimum of four pictures were quantified and averaged per sample.

Cellular viability was further validated by using the 3-(4,5-dimethylthiazol-2-yl)-2,5-diphenyltetrazolium bromide (MTT) assay (ROCHE, Sigma-Aldrich) according to the manufacturer’s instructions. Briefly, MTT labeling reagent (final concentration 0.5 mg/mL) was added to each well and incubated for 4 h in a humidified atmosphere (37 °C, 5% CO_2_). Then the solubilization buffer was added to the reaction and incubated overnight. After total solubilization of the purple formazan crystals, samples were collected in three replicates in a 96-well plate. The absorbance signal (wavelength: 580 nm, reference wavelength: 650 nm) was measured in a TECAN Infinite M200 Pro microplate reader.

### Cell Adhesion and Morphology

Cellular morphology and attachment were further monitored throughout the culture period, and phase contrast images were taken using a Leica DM IL LED fluorescence microscope. Similarly, immunostaining images of cellular actin (Phalloidin-TRITC, Sigma-Aldrich) were taken at different time points to observe cellular morphology using a wide-field microscope (Leica Thunder 3D Live Cell).

After 7 days of culture, cells were fixed with 2.5% glutaraldehyde in phosphate buffer. Fixed samples were then washed four times and dehydrated by immersion in increasing alcohol concentrations. Next, the specimens were dried by critical point drying and mounted on a metal stub using a sticky carbon disc tape to increase conductivity. Samples were then coated with an ultrathin gold layer and observed in a field emission scanning electron microscope (NOVA NanoSEM 230, FEI Company, IBEC).

### H9c2 Differentiation

H9c2 cells were seeded at a density of 30,000 cells/cm^2^ and maintained in a proliferative state overnight by culturing in growth media (high-glucose DMEM supplemented with 10% FBS, 1% l-glutamine, and 1% penicillin/streptomycin) at 37 °C in a humidified atmosphere of 5% CO_2_. Differentiation was performed by adapting previously described protocols.^45,46^ Briefly, cell growth medium was changed to differentiation medium (high-glucose DMEM (same supplementation) using 1% FBS and 10 nM retinoic acid (Sigma) supplementation to improve cardiomyocyte yield). Culture in this medium was continued for at least 6 days with daily media changes in the dark. All-trans-RA was prepared in DMSO and stored at −20 °C in the dark to avoid degradation.

### Western Blot

Protein expression of cardiomyocyte markers was studied by Western Blot. Protein samples were prepared by lysing cells with Pierce RIPA Buffer (Thermo Fisher) supplemented with a protease inhibitor cocktail (Abcam) for 10 min shaking on ice. Then samples were centrifuged for 10 min at 4 °C at 10,000g, and the supernatant was collected and stored at −80 °C until analysis. Total protein content was measured with a Pierce BCA Protein Assay Kit (Thermo Scientific) following the manufacturer’s instructions. Equal protein amounts were mixed with Laemmli Sample Buffer (Bio-Rad) and denatured at 95 °C for 5 min. Samples were loaded in 10% Mini-PROTEAN TGX Precast Protein Gels (Bio-Rad) and run at constant amperage (20 mA/gel) for 1–2 h. Protein samples were transferred onto a nitrocellulose membrane (Bio-Rad) by wet transfer at 100 V for 1 h at 4 °C. Ponceau staining was used to assess a correct protein transfer. Membranes were blocked in Tris-buffered saline containing 0.1% Tween 20 (TTBS) with 5% skim milk for 1 h at room temperature and incubated overnight with primary antibodies at 4 °C. Antibodies against Cardiac Troponin T (sc-20025 mouse monoclonal antibody, Santa Cruz, 1:1000), Connexin-43 (ab11370 rabbit polyclonal antibody, Abcam, 1:2000), and Heavy Chain Cardiac Myosin (ab50967 mouse monoclonal antibody, Abcam, 1:1000) were employed. GAPDH (PA1–987 rabbit polyclonal, Thermo Fisher, 1:2000) and Vinculin (V9131, Merck, 1:200) were used as loading controls to normalize the data. Horseradish peroxidase-conjugated antibodies against rabbit (ab97051, Abcam, 1:10,000) or mouse (ab97023, Abcam, 1:5000) were used in TTBS-5% skim milk solution and incubated for 1 h at room temperature. Membranes were developed using Clarity Western ECL Substrate (Bio-Rad), and the chemiluminescence was detected in a densitograph (AS4000 Imaging System, GE Healthcare Life Sciences). Quantitative analysis was performed using ImageJ software.

### Immunostaining

Following culture, cells were washed in PBS and fixed in 4% formaldehyde solution (Electron Microscopy Sciences) for 10 min at room temperature. Fixed cells were then washed in cold PBS with 0.15% glycine (Sigma) three times, permeabilized in 0.05% Triton X-100 (Sigma) in PBS-0.15% Gly for 10 min, and blocked in PBS-0.15% Gly-5% BSA solution for 45 min at room temperature. Cells were incubated with the corresponding primary antibody (Cardiac Troponin T (sc-20025 mouse monoclonal antibody, Santa Cruz, 1:100), Ki67 (ab16667 rabbit monoclonal antibody, Abcam, 1:250), Ryanodine R Receptor (ab219798 rabbit monoclonal antibody, Abcam, 1:100)) in PBS-0.15% Gly-1% BSA at 4 °C overnight. Then cells were washed three times for 5 min in PBS-0.15% Gly. Samples were then incubated with the corresponding secondary antibody in PBS-0.15% Gly-1% BSA for 1 h at room temperature in the darkness (goat antimouse Alexa Fluor 488 ab150113, 1:500 and donkey antirabbit Alexa Fluor 647 ab150075, 1:500). Cells were then washed three times, counterstained with 4′,6-diamidino-2-phenylindole (DAPI, Sigma, 1:500) and actin (Phalloidin-TRITC, Sigma-Aldrich P1951, 3.5:500) for 30 min, washed three times again, and stored in PBS at 4 °C until imaged. Immunolabeled samples were imaged using a wide-field microscope (Leica Thunder 3D Live Cell). At least four images per sample were analyzed. Quantitative image analysis was performed using ImageJ software.

### Statistical Analysis

Data are presented as mean and error bars representing standard deviation (SD) from biological replicates. GraphPad Prism 9.4 was used as analysis and graphical software. Student’s *t* test (two-tailed distribution) was generally used to compare two samples, one-way ANOVA followed by *post hoc* Tukey’s test was used for multiple samples, and two-way ANOVA followed by *post hoc* Tukey’s test was used when two different categorical independent variables were tested for multiple samples. A *p*-value < 0.05 was considered statistically significant. Significance was represented as follows: *p < 0.05, **p < 0.01, ***p < 0.001, ****p < 0.0001.

## Results and Discussion

### BC-Ppy Synthesis

Bacterial cellulose (BC) films were naturally produced by *K. xylinus* bacteria, extracellularly, at the medium–air interface. In brief, the monomer (pyrrole) was added to a solution of PVA + FeCl_3_·6H_2_O in the presence of BC films. Here, the FeCl_3_·6H_2_O was used as an oxidant-cum-dopant providing the dopant anion Cl^–^, whereas PVA acts as a biocompatible surfactant controlling the size of Ppy NPs and preventing their aggregation (Figure S2A).

**Figure 1:**
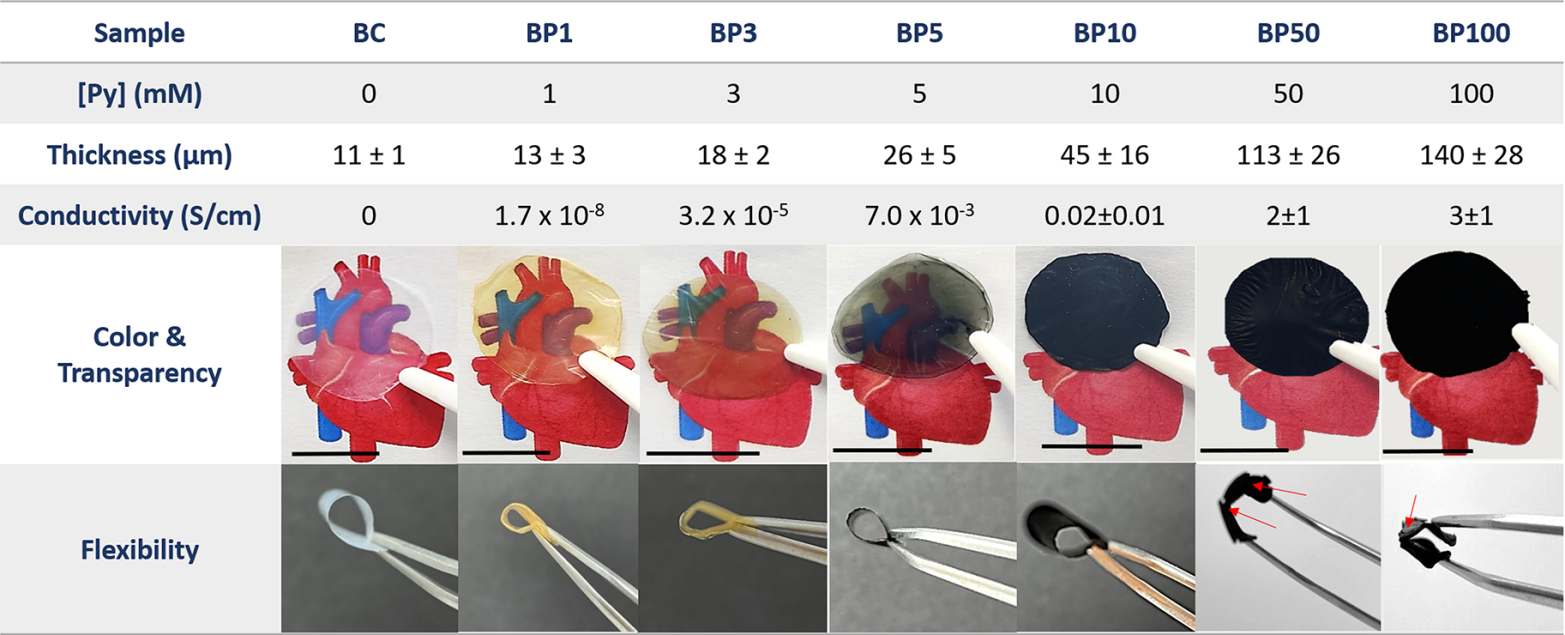
Effect of Py concentration on scaffolds’ properties. Bacterial nanocellulose (BC) and bacterial nanocellulose-polypyrrole nanocomposites (BC-Ppy) at different concentrations of pyrrole (Py) and their change of properties. Scale bars: 1 cm.

The composition and properties of BC-Ppy composites can be easily tuned by modifying the precursor concentration. Hence, we prepared BC-Ppy using different starting Py monomer concentrations from 1 mM to 100 mM. BC films changed color from white (transparent) to yellow–dark-green–black (opaque) color, reducing the transparency with increasing monomer concentrations (Figure 1). The thickness of the scaffolds also increased after Ppy functionalization, reaching up to ∼140 μm at the highest concentration (100 mM Py) in the dry state, whereas the initial dried BC films were ∼11 μm in thickness (Figure 1). BC-Ppy composites maintained the inherent flexibility of BC up to 10 mM Py concentration. High Ppy content fragilized the composite, and some breaks were observed upon bending (see Figure 1 and red arrows).

The BC-Ppy composites synthesis is simple and highly versatile. We used a commercial BC scaffold, cube of 1 cm, to incorporate Ppy and create a thick 3D structure. Ppy NPs were incorporated homogeneously within the 3D structure as the same color was observed in the exterior and the cross-section of the cube (Figure S2B), confirming the capability of extending to different BC structures.

### Size and Morphology

**Figure 2:**
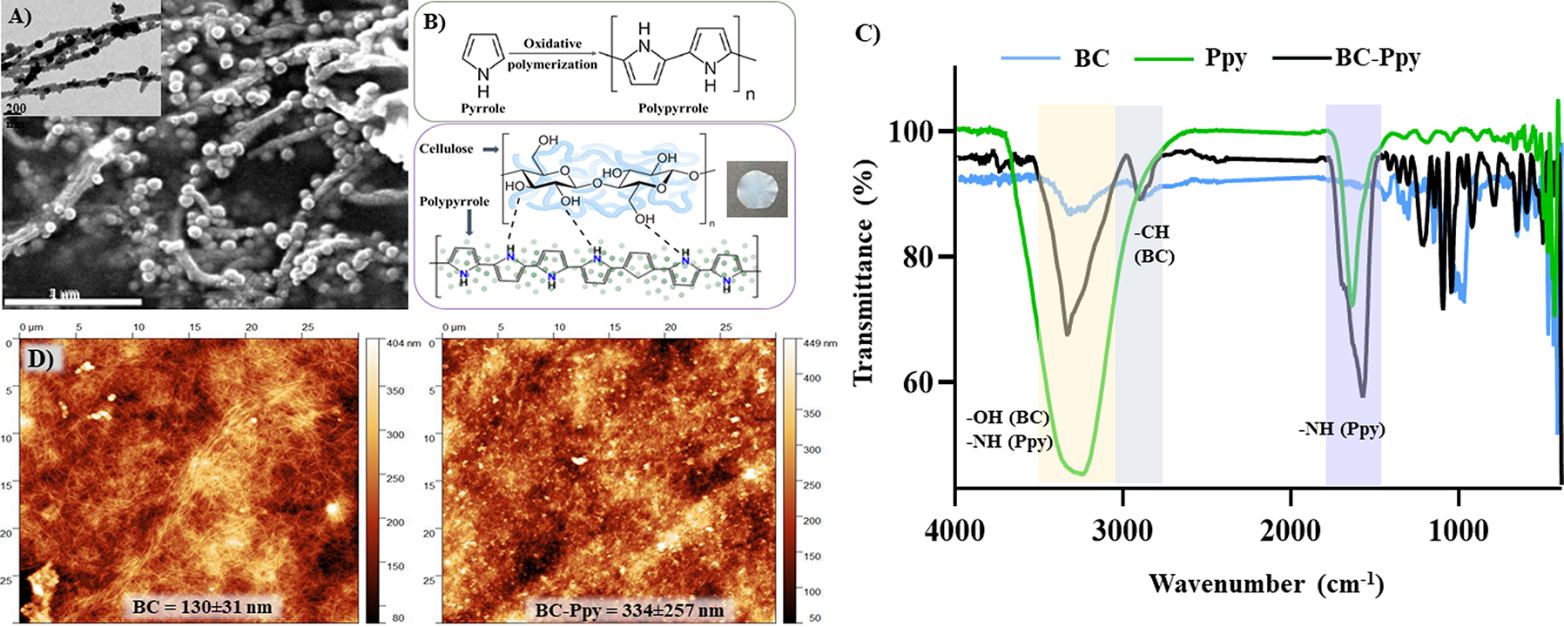
Characterization of BC and BC-Ppy composites. A) SEM and TEM (inset) of BC-Ppy powders showing size, morphology, and distribution. B) Polypyrrole formation reaction and interaction between BC and Ppy. C) FTIR spectra. D) AFM images of BC and BC-Ppy showing surface roughness. Scale bar: 1 μm.

At the micron scale, scanning electron microscopy (SEM) of BC-Ppy revealed that Ppy NPs had a spherical shape and were homogeneously distributed along the BC fibers (Figure 2A). The diameters of Ppy NPs and BC fibers were computed from TEM images and found to be 83 ± 8 and 65 ± 12 nm, respectively (Figure S3). Decoration of BC fibers with Ppy NPs does not affect the fiber diameter compared to plain BC. The composites exhibited fibers of uniform diameter throughout the films, possessing branches displaying a random mesh-like structure, similar to the ECM^30^ (Figure 2A). It is also worth noting that, despite severe sample processing of the composites (involving blending, drying, and grinding), the NPs are still bound to the fibers, suggesting a strong interaction between the two components (Figure 2B).

### Chemical Properties

The FTIR spectra of BC, Ppy NPs, and BC-Ppy composites are shown in Figure 2C. Spectra presented a peak around 3326 cm^–1^ corresponding to the −OH vibrations of BC, whereas the peak at 3249 cm^–1^ is assigned to the Ppy aromatic secondary amine (N–H) stretching. This latter peak shifted to 3345 cm^–1^ for the BC-Ppy composites, indicating a plausible hydrogen bonding between the −NH group of Ppy and the −OH group of cellulose^35^ (Figure 2B). The peak at 2956 cm^–1^ corresponds to the aliphatic glycosidic CH bonds of cellulose BC-Ppy composites. PVA peaks at ∼1409 cm^–1^ assigned to C–H bending were not identified in the FTIR, indicating that the PVA amount is low and any excess was efficiently removed by the cleaning of the samples.^47^ On the other hand, the strong peak at 1635 cm^–1^ found in the composites confirmed the existence of N–H bending. The N–H bending peak also shifted to 1592 cm^–1^ in the composites, again pointing to hydrogen bond interactions. These observations are consistent with previously published reports on BC and Ppy composites.^35,48−51^ It is well-known that BC has an abundant hydroxyl (−OH) group and Ppy has a secondary amine group in its repeating unit^52,53^ (Figure 2B). Therefore, based on the literature, we suggest that these groups form strong hydrogen bonds. The uniform distribution of −OH groups within BC and −NH groups within Ppy facilitated the homogeneous distribution of the NPs along the fibers as observed in the SEM and TEM images.

Atomic force microscopy (AFM) also confirms the presence of Ppy NPs and indicates that the surface roughness increased with the presence of Ppy NPs from 130 ± 31 nm for plain BC to 334 ± 257 nm for the BC-Ppy composites (Figure 2D). This effect is clearly related to the Ppy NPs attached to the fibers, which could also act as an anchoring point of cells as reported previously with BC-TiO_2_ NPs.^31^

### Electrical Properties

**Figure 3:**
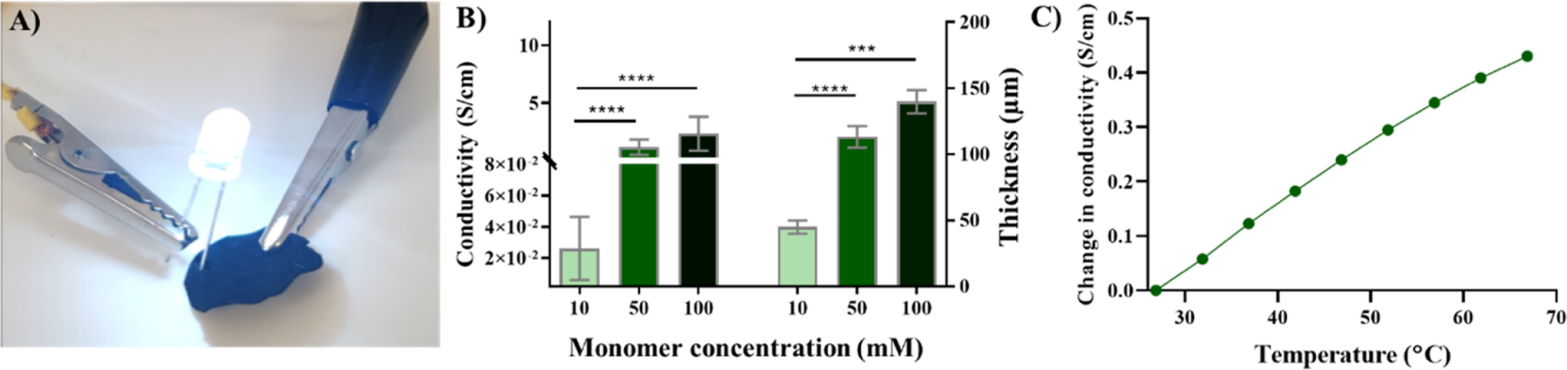
Conductivity of BC-Ppy films. A) Conductivity of BC-Ppy films shown by a simple LED setup. B) Effect of initial monomer concentration in conductivity and thickness. C) Effect of temperature on conductivity.

BC-Ppy scaffolds were conductive, and we proved it using a simple LED setup, with the two probes placed on two ends of a dried BC-Ppy film. The LED bulb lights up upon contact with the surface (Figure 3A). Ppy exhibits a wide range of conductivity (from 10^–10^ to 10^4^ S/cm) depending on its doping level, type of dopant, the extent of polymerization, etc.^29^ Consequently, the electrical conductivity and thickness of BC-Ppy films as a function of Py monomer concentration were assessed. The thickness of BC-Ppy composites increased with pyrrole monomer concentration (Figure 3B), which can be attributed to the increased number of Ppy NPs attached to BC fibers improving the connectivity between Ppy domains. We prepared BC-Ppy with 1, 5, 10, 50, and 100 mM Py concentrations, and the average conductivity of BC–Ppy materials were 1.7 × 10^–8^, 3.2 × 10^–5^, 7 × 10^–3^, 0.02 ± 0.01, 1.7 ± 1.3, and 2.4 ± 1.5 S/cm, respectively (Figure 3B, Figure 1). We evaluated the conductivity of BC-Ppy as a function of temperature and found that temperature increased the conductivity. For instance, at 100 mM monomer concentration, BC-Ppy showed 0.1 S/cm higher conductivity at 37 °C than at room temperature (Figure 3C), since in organic semiconductors, charge carrier transport occurs through hopping, which is a thermally activated mechanism.^54^ Therefore, when the scaffolds are employed for cell culture at 37 °C, they are expected to exhibit a slightly higher conductivity than what was measured at room temperature. Previous works reported Ppy materials reaching conductivities larger than 380 S/cm and applying them in purely electronic applications such as sensors, microelectronic devices, supercapacitors, etc.^29,55,56^ The conductivity obtained in our BC-Ppy films, using FeCl_3_·6H_2_O as the oxidant and Cl^–^ anion doping, was higher than published reports of BC-Ppy composites (10^–4^ S/cm for 0.05 M Py) using ammonium persulfate as the oxidant with sulfate ions as anionic dopant.^37^ Interestingly, the type of oxidant used for the dopant ion also plays a significant role in influencing conductivity. Another study of BC-Ppy composites with the same dopant anion (Cl^–^) for neural tissue engineering by Thunberg *et al.* reported lower conductivities (10^–4^ S/cm for 0.1 M Py) than us for the same monomer concentrations.^39^ For the following evaluation of mechanical properties and cell experiments, we chose BC materials prepared with 1–10 mM Py concentrations (hereafter referred to as BP1 (1 mM), BP3 (3 mM), BP5 (5 mM), and BP10 (10 mM)), which exhibited conductivity between 10^–8^ and 10^–2^ S/cm since cardiac TE applications do not call for high conductivities^38,57^ given that the electrical conductivity of myocardial tissue is ≈10^–3^ S/cm.^58,59^

### Stability of BC-Ppy

Material’s conductivity was not affected by UV sterilization, storage in water, or incubation in cell culture media (Figure S4A). However, although non-significant, a decrease in conductivity could be observed after autoclaving (Figure S4A). This is possibly due to the overoxidation of Ppy at higher temperatures and diffusion of the dopant Cl^–^ ions into the solution.^57^ Therefore, in this study, the composites were always sterilized by UV exposure prior to cell-culture experiments. We also evaluated any possible leaching of Ppy NPs into the solution upon storage in water, sterilization, and incubation in the cell-culture media by UV–vis/NIR spectroscopy. No leaching of Ppy NPs was observed, indicating that the experimental conditions of the cell-culture studies do not cause any leaching from the scaffold (Figure S4B), and they are safe to be employed.

### Mechanical Properties

**Figure 4:**
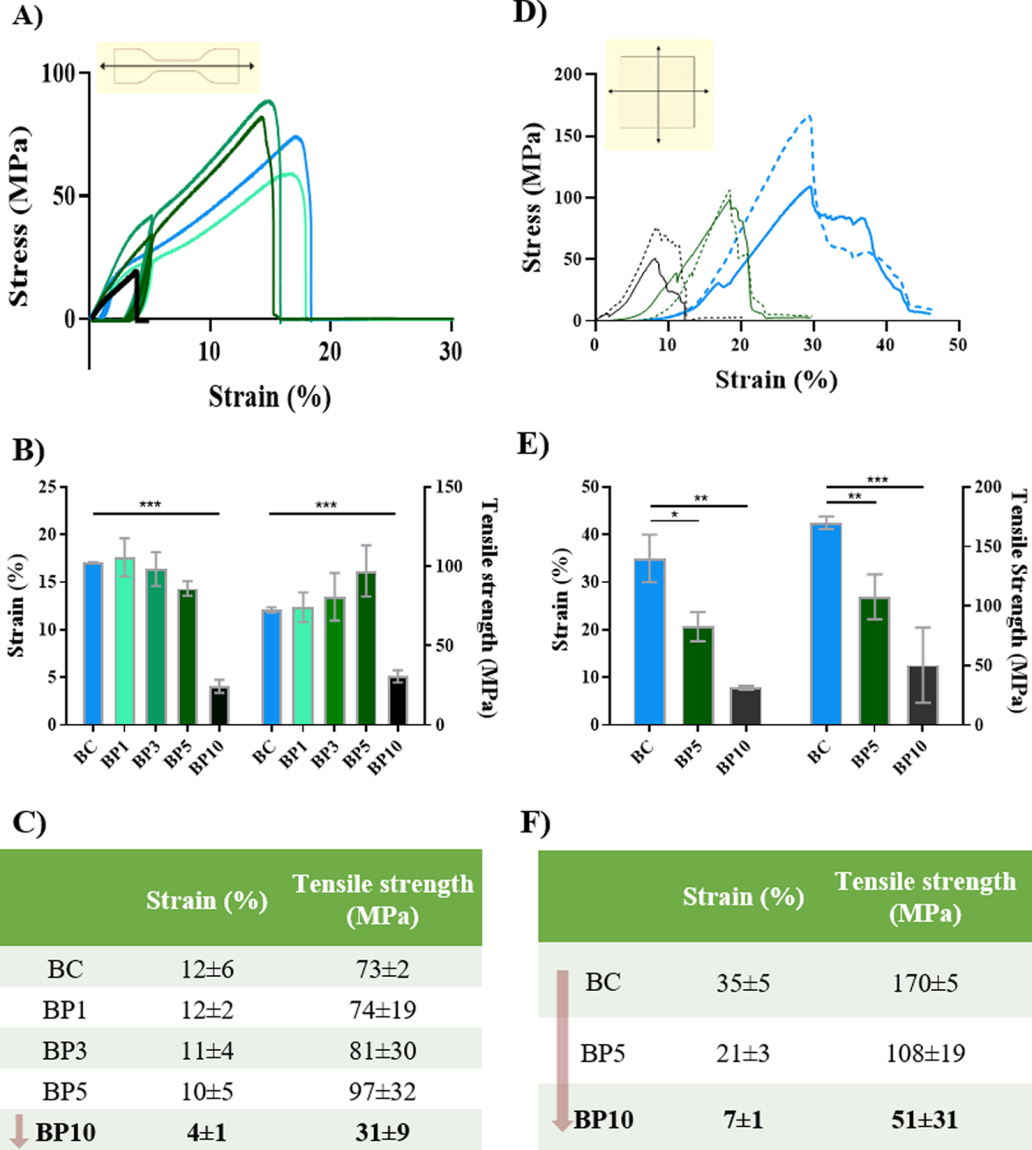
Mechanical properties of BC and BC-Ppy scaffolds. A) Uniaxial tensile test stress–strain curve. B,C) Breaking strain % and tensile strength from uniaxial tensile test. D) Biaxial stress–strain curve (solid lines–force along *x*-axis; dashed lines–force along *y*-axis). E,F) Breaking strain % and tensile strength from biaxial tensile test.

Cardiac tissue withstands repetitive cyclic loading due to cardiac beating. Native myocardial tissue exhibits tensile strengths ranging from 0.4 (pulmonary artery) to 2.6 MPa (native circumferential heart valve) at 22% tensile strain under uniaxial cyclic tensile loading.^60^ We performed cyclic uniaxial tensile tests to reproduce the repetitive beating of the heart. The tensile strength of BC-Ppy materials increased slightly with the Ppy content, with a concomitant decrease in breaking strain. However, BP10 (with the highest Ppy content) possessed a tensile strength as low as ∼31 MPa and failed at ∼4% strain (Figure 4A–C). This indicates that adding Ppy decreases the tensile strain of the composites, making them more brittle, especially pronounced at concentrations above 5 mM.

We also performed multiaxial tensile tests to get a deeper insight into the mechanical properties of BC, BP5 and BP10 scaffolds (Figure 4D–F), since the myocardium is a tissue with anisotropic mechanical behavior.^61,62^ Tensile strength and breaking strain decreased as polypyrrole concentration increased, similar to the uniaxial results (Figure 4F). Interestingly, materials exhibited a more elastic behavior and higher tensile strength (108 MPa for BP5) and strain at failure (20% for BP5) under biaxial stress compared to uniaxial. This is possibly due to the material experiencing uniform strain from all directions, creating symmetrical force, and making it more difficult to reach a breaking strain in a particular direction, which is probably conferred by the BC scaffold. In both uniaxial and biaxial stress, BP10 has the lowest tensile strength and ductility (Figure 4). Overall, the mechanical strength of BC-Ppy composites decreased compared to plain BC, substantiating the strong interaction via H-bonding, which could influence the interaction with cells.

### Surface Wettability

**Figure 5:**
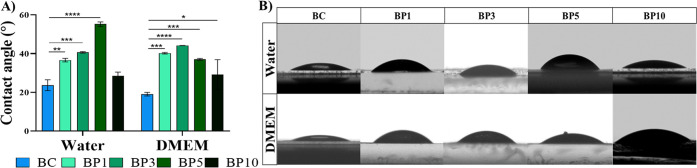
Surface wettability of BC and BC-Ppy. A) DMEM and water contact angle measurements of BC and BC-Ppy composites. B) Sample pictures of water and DMEM droplets on the scaffold surfaces.

The surface wettability of materials influences cell adhesion and in turn, biocompatibility. We measured the contact angle in water and the cell culture media DMEM for all scaffolds (BC, BP1–BP10). All the BC and BC-Ppy materials displayed hydrophilic behavior, with contact angles < 90°, which increases at higher Ppy content, indicating a higher hydrophobicity (Figure 5). Beyond the BP5 in water and BP3 in DMEM, the contact angle starts to decrease. The lower contact angle with increased Ppy content can be attributed to a high doping level of hydrophilic Cl^–^ ions and also to higher coverage of the BC-Ppy. In a nutshell, Ppy NPs on BC materials increase the hydrophobicity of the scaffolds, which is also substantiated by the increased surface roughness and amount of NPs in the materials.

### Viability of Cardiac Cells on BC-Ppy Scaffolds

Based on our characterization, we selected BC-Ppy scaffolds with low Ppy content (0, 1, 3, 5, and 10 mM) for the subsequent cellular studies since these concentrations have conductivities in the range of cardiac native tissue.^58,59^ The cytocompatibility of BC-Ppy composites was examined considering the most important cardiac cell types: cardiomyocytes, which generate contractile forces and regulate rhythmic heart beating,^63^ and cardiac fibroblasts, the largest cell population of the heart and key in the remodeling process occurring after heart injury.^64^ Consequently, the viability of H9c2 cardiomyoblasts and human adult cardiac fibroblasts (HCFs) on BC-Ppy scaffolds have been investigated.

**Figure 6:**
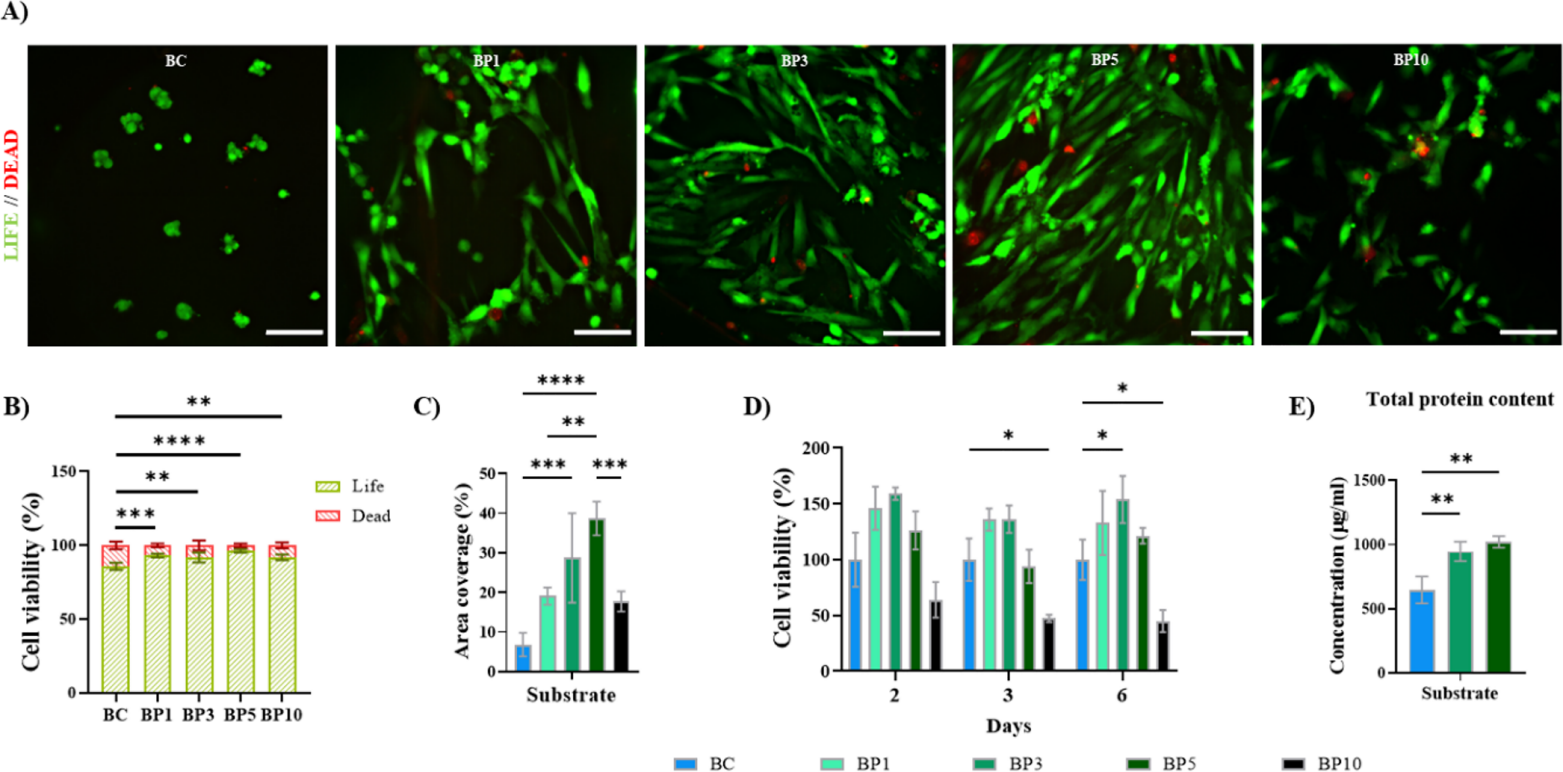
Viability of H9c2 onto BC and BC-Ppy scaffolds. A) LIVE/DEAD Staining of H9c2 cells cultured for 24 h onto BC and BC-Ppy scaffolds. B) Quantification of LIVE/DEAD images (*n* = 4). C) Area coverage of cells adhered to BC and BC-Ppy scaffolds after 24 h of culture (*n* = 4). D) Cytotoxicity of H9c2 cardiomyoblasts after 2, 3, and 6 days of culture analyzed by MTT (*n* = 3 at day 2 and *n* = 4 at days 3 and 6; data normalized to the absorbance values obtained in plain BC materials at each time point). E) Quantification of the total protein content of H9c2 cells grown onto BC, BP3, and BP5 after 7 days of culture (*n* = 4). Scale bars: 100 μm.

The cytocompatibility of BC–Ppy composites was characterized by culturing H9c2 cells and evaluated by LIVE/DEAD staining. The number of live cells was quantified after 24 h of culture, and all the scaffolds exhibited excellent cytocompatibility for H9c2 cells. Adding Ppy further increased the percentage of alive cells (Figure 6A,B), suggesting that the electro-conductive properties of BC-Ppy composites improve the cytocompatibility of BC scaffolds. In fact, Ppy has been previously blended with a wide variety of inert biomaterials, and the resulting Ppy-based constructs showed exceptional biocompatibility with cardiac cells.^65−70^ The presence of Ppy enhanced the adhesion of H9c2 cells onto the composites compared to plain BC scaffolds (∼7% cell area coverage), reinforcing the beneficial effect of Ppy (Figure 6A–C). Among all tested BC-Ppy materials, BP5 rendered the best results in terms of cell attachment, reaching a cell coverage of ∼40%. BC-Ppy composites with higher Py concentrations (BP10) exhibited a decrease in the number of adhered cardiomyoblasts, even though they still outperformed plain BC scaffolds. The reduced H9c2 cells’ density in BP10 can be attributed to the higher concentration of Ppy in BP10 composites, which could be deleterious to H9c2 cells. Indeed, Ppy has been previously shown to be cytotoxic when used at elevated concentrations.^68^

The bioactivity of H9c2 cells on BC and BC-Ppy scaffolds was further examined postseeding at days 2, 3, and 6 by MTT assay. We found improved cell growth in the presence of Ppy (Figure 6D). BP1, BP3, and BP5 scaffolds outperformed BC with increased proliferation of H9c2 cells at all time points. The bioactivity of BC and BC-Ppy scaffolds was also evaluated when used in their “wet” form and rendered akin results (Figure S5). We confirmed this increase in H9c2 proliferation by quantifying total protein by bicinchoninic acid (BCA) assay after 7 days of culture (Figure 6E). The significant increment of total protein in BC-Ppy scaffolds suggests an increase in the total number of cells, which further confirms our previous findings: Ppy addition enhances cell adhesion to BC scaffolds.

Similarly, the cytocompatibility of our composites was tested with HCFs. Excellent viability and adhesion of HCFs onto both BC and BC-Ppy substrates were reported, even at higher concentrations of Ppy (see detailed information in Figure S6). All in all, BC-Ppy composites are cytocompatible with cardiac cells. Adding Ppy (up to 5 mM) to BC significantly enhances cell viability, growth, and adhesion of H9c2 cardiomyoblasts to the materials. On the other hand, HCFs grow and adhere better to plain BC substrates and can withstand higher concentrations of conductive Ppy in comparison to H9c2 cells, showing a less sensitive behavior than cardiomyoblasts. Previous work of Gelmi *et al.*(70) also described distinct sensitivities among different cardiac cell types. They tested endothelial progenitor cells (EPCs) and cardiac progenitor cells (CPCs) onto different Ppy-containing substrates. While all Ppy (dopant) materials were biocompatible for EPCs, CPCs showed sensitivity toward some of the biomaterials and displayed significantly decreased cell viability and density.^70^

It has been reported that nanocellulose-Ppy composites need preconditioning (extensive rinsing and preincubation in a biological buffer for 48 h) to obtain a noncytotoxic biomaterial.^71^ Therefore, we lastly evaluated the potential cytotoxicity of nonpreconditioned BC and BC-Ppy scaffolds. For this, BC/BC-Ppy composites were incubated overnight with cell media to allow leakage, if any, of materials’ components into the medium. Then, medium was collected and added to H9c2 cells. No toxic effect was reported by any of our as-synthesized scaffolds (Figure S7), indicative of a superior methodology to obtain nontoxic nanocellulose-Ppy composites in comparison with previously described methods^71^ in which additional steps are needed to ensure good cell cytocompatibility.

### H9c2 Cardiomyoblasts Attachment and Morphology on BC-Ppy Scaffolds

**Figure 7:**
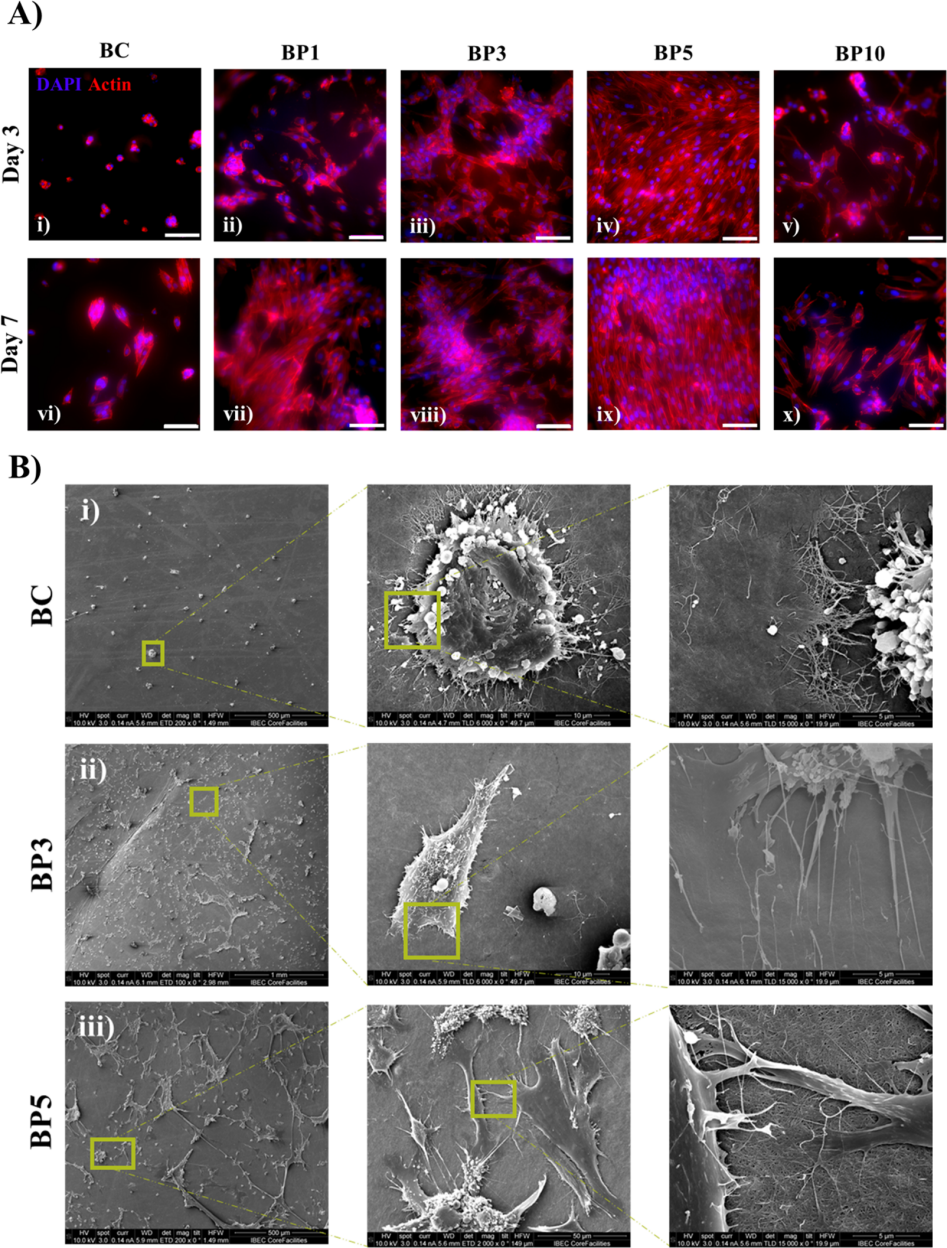
H9c2 attachment to BC and BC-Ppy scaffolds. A) Fluorescence microscopy images of H9c2 cell populations seeded on nanocellulose composites after 3 and 7 days of culture. Scale bars: 100 μm. B) SEM images depicting H9c2 attachment and morphology after 7 days of culture onto BC, BP3, and BP5 materials.

Cell morphology of H9c2 was examined on days 3 and 7 of culture. Cardiomyoblasts did not properly attach to plain BC. Indeed, a few cells adhered to BC materials displaying an atypical round shape and gathering in small clusters (Figure 7A i, vi). The addition of polypyrrole to BC scaffolds promoted the attachment of a higher number of cells, and adhered cells retained the characteristic H9c2 spread-out morphology^45^ (Figure 7A ii-v, vii-x). In line with previous findings (Figure 6A–C), BP5 scaffolds were the best-performing ones. Cardiomyoblasts seeded on BP5 scaffolds showed similar cell density and morphology to controls (Figure S8). Other time points were imaged using phase contrast and showed the same trend (data not shown). Similarly, H9c2 cells grown onto “wet” BC-Ppy materials exhibited an identical behavior (Figure S9).

Cell–scaffold interactions were also examined after 7 days of culture by scanning electron microscopy (SEM). For this, only BP3 and BP5 were investigated as guided by previous experiments. SEM images showed that cells on BC-Ppy produce long filopodia that extend to the scaffolds, while cells on BC scaffolds exhibited limited filopodia. The lower H9c2 density and the uncharacteristic round cell shape of cardiomyoblasts on plain BC materials, which we reported previously by other methods, were confirmed. Cell bodies did not fuse well to BC scaffolds (Figure 7B i). Nevertheless, H9c2 cells elongated and attached to BC-Ppy materials with higher extension, adhesion, and cell density. The addition of Ppy to the BC scaffold allowed H9c2 cells to interact with the BC-Ppy nanofibers and cardiomyoblasts amalgamated with the BC-Ppy materials (Figure 7B ii, iii).

Cellular morphology serves as an indicator of cellular state and functionality.^72^ In brief, H9c2 exhibited an abnormal cell shape when grown onto BC scaffolds, and the addition of Ppy allowed cells to retain their characteristic cell morphology. These results suggest that Ppy may promote a cardiac phenotype given its biomimetic conducting properties. Indeed, existing evidence shows a positive influence of electroconductive biomaterials *in vitro* including improved cytoskeletal organization, myocardial tissue maturation, or synchronization of cell beating, among others.^23,73^

### Effects of Conductive BC-Ppy Scaffolds on H9c2 Differentiation *In Vitro*

H9c2 is a myoblast cell line derived from embryonic rat ventricular heart tissue.^74^ H9c2 cells have been extensively used in cardiovascular research as an alternative for adult primary cardiomyocytes (PCMs).^45,46,75^ PCMs are ideally the supreme cellular model for cardiovascular basic research;^76,77^ however, their use is extremely limited by the technical intricacies in their isolation, culture, and scale production.^78^ Despite the latest attempts to optimize PCM isolation, unstable quality and low cell yield are major hurdles that preclude the use of PCMs in nonacute *in vitro* experiments.^78,79^ In this scenario, H9c2 surpasses other existing cardiac cell lines (e.g., HL-1, AC16)^76,80^ and can be further matured toward a cardiomyocyte-like phenotype when treated with retinoic acid (RA).^45,46^ However, it was brought to our attention that recent studies using H9c2 cells to test distinct biomaterials for cardiac applications did not induce such a cardiomyocyte differentiation,^40,81−84^ suggesting plausible difficulties to mature H9c2 cells when grown onto engineered substrates. Additionally, other laboratories reported discrepancies when reproducing H9c2 maturation to cardiomyocytes,^76,85^ indicating some degree of resistance to differentiation. Nevertheless, H9c2 differentiation is imperative to achieve a better resemblance to adult cardiac tissue and perform investigations with higher translational value. Consequently, in the present study, we aim to characterize the behavior and differentiation capabilities of H9c2 cardiomyoblasts grown onto BC-Ppy composites.

We first confirmed the capabilities of H9c2 maturation to cardiomyocytes upon differentiation treatment. Two differentiation media were tested (containing 10 nM and 1 μM RA) and efficiency was assessed after two treatment durations (6 and 10 days). H9c2 differentiation was confirmed at both time points by using any of the differentiation media (see detailed information in Figure S10). Six days of treatment and differentiation media containing 10 nM RA were employed henceforth.

**Figure 8:**
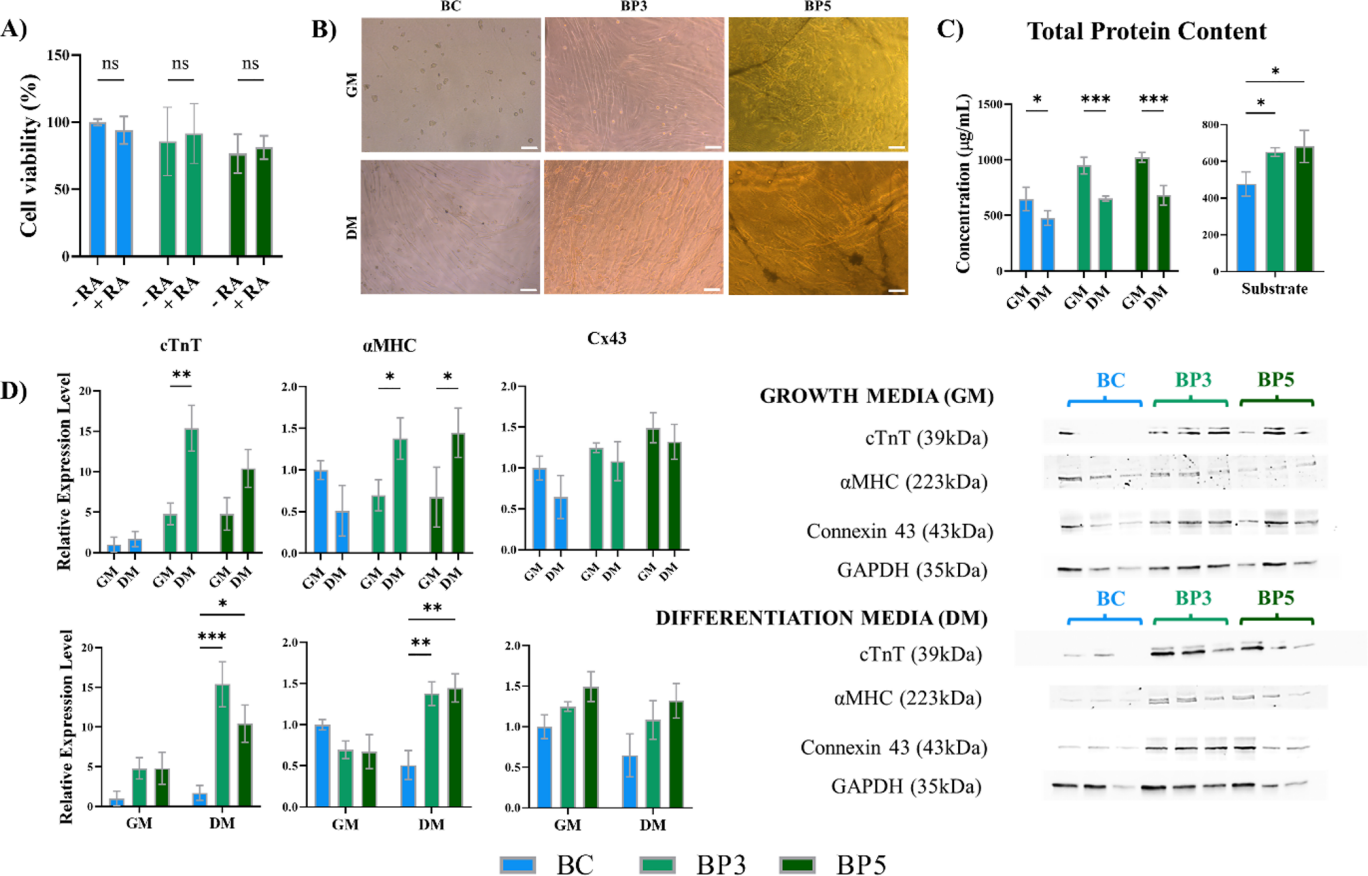
H9c2 differentiation onto BC/BC-PPy scaffolds. A) Combined toxicity of RA with BC/BC-Ppy byproducts on cardiomyoblasts (*n* = 4, data normalized by viability of H9c2 cells incubated with growth medium (without RA) from plain BC scaffolds). B) Morphological change of cardiomyoblasts grown onto BC/BC-Ppy materials upon differentiation treatment. C) Left: Total protein content of H9c2 grown onto different substrates with growth and differentiation media after 7 days of culture (*n* = 3). Right: Quantification of total protein content of H9c2 cells grown onto BC, BP3, and BP5 with differentiation treatment (*n* = 3). D) Protein expression of specific cardiac markers cTnT, αMHC, and Connexin 43; GAPDH was used as the loading control (*n* = 3, protein expression data normalized to intensity values of cells grown onto plain BC materials with growth medium). GM = growth medium; DM = differentiation medium. Scale bars: 100 μm.

Differentiation media contains RA; consequently, the possible combined toxicity of RA with the BC/BC-Ppy byproducts was evaluated. Growth medium with and without 10 nM RA was incubated with the BC/BC-Ppy composites. After overnight incubation, media was collected and added to H9c2 cells for 24 h before checking cell viability by MTT. The combination of BC/BC-Ppy byproducts with RA did not affect H9c2 cells viability (Figure 8A).

We then proceeded to chemically differentiate H9c2 cells onto BC-Ppy composites. H9c2 morphologic alterations were observed by phase contrast microscopy after differentiation treatment. H9c2 cells cultured in a growth medium retained their characteristic shape when grown onto BC-Ppy substrates, whereas they showed an atypical, rounded morphology when grown onto BC scaffolds, as previously described (Figure 8B, top panel). After culture with differentiation media, H9c2 myoblasts fused and showed an elongated shape in all conditions (Figure 8B, bottom panel). The acquisition of such a morphological signature evidence the successful chemical differentiation of H9c2 cells when grown onto bacterial cellulose composite substrates, which, to the best of our knowledge, was not reported until now.

As expected, inducing H9c2 differentiation onto the composites reduced cell proliferation (Figure 8C, left). Nevertheless, even under differentiation treatment, the presence of Ppy significantly increased cell growth as evidenced by the increment in total protein (Figure 8C, right). This is in line with our previous results (Figure 6) and suggests that the reported beneficial effects of Ppy in H9c2 cardiomyoblasts are preserved as well in differentiated cardiomyocyte-like H9c2 cells.

Given that functional assays are limited in H9c2 due to their inability to beat,^75^ differentiation of H9c2 cells onto the different substrates was further confirmed by the increased expression of cardiac-specific markers, indicating a cardiomyocyte-like phenotype. Higher cardiac troponin T (cTnT) expression was observed when H9c2 were cultured with differentiation media (DM) in all conditions. Similarly, alpha myosin heavy chain (αMHC) expression increased upon treatment, although exclusively when BC-Ppy composites were employed. No differences in the expression of the gap junction protein Connexin 43 (Cx43) were seen (Figure 8D, top graphs).

Interestingly, H9c2 differentiation into cardiomyocytes was promoted by solely adding polypyrrole to the BC substrates (Figure 8D, bottom graphs). cTnT expression increased when H9c2 cells were grown onto BC-Ppy materials by using standard growth medium (GM) (i.e., without inducing myoblast differentiation chemically). Additionally, when H9c2 maturation was induced with differentiation media (DM), higher expression of cTnT was seen in cells grown onto BC-Ppy composites in comparison with plain BC scaffolds. αMHC expression only increased when H9c2 cells were cultured with differentiation media and grown onto Ppy-containing composites, suggesting a combined effect. In fact, αMHC content was also characterized in control cultures (material-free well plates) and its expression levels did not change by solely inducing differentiation chemically (Figure S11), reinforcing a possible synergistic effect of Ppy and the differentiation media. Lastly, Cx43 expression increased with Ppy concentration when BC-Ppy materials were used as substrates, regardless of the use of differentiation media or not.

In a nutshell, we found that adding the electrically conductive polymer polypyrrole to BC biomaterials fosters H9c2 maturation *in vitro*. Similarly, other polypyrrole-based conductive constructs have been reported to enhance the functional properties of cardiac cells, reinforcing our findings to a greater extent. Spearman *et al.* reported enhanced Cx43 expression and improved Ca^2+^ transients in cardiomyocytes when Ppy was added to their polycaprolactone matrix.^66^ Tsui *et al.* showed enhanced cellular organization, sarcomere development, and improved expression of cardiac markers (Cx43, Myh7, SCN5A, cTnT) when conjugating Ppy to acid-modified silk fibroin cardiac scaffolds.^69^ Comparably, Gelmi *et al.* proposed a novel Ppy-containing electromechanically active composite to promote differentiation of hiPSCs to cardiomyocytes.^86^ Coating their fibrous scaffolds with Ppy increased the expression of cardiomyocyte-specific genes (Actinin, Myh6) and regulators of cardiac differentiation (NKX2.5, GATA4). Nevertheless, they used preconditioned hiPSCs (3-days culture with differentiation medium prior to cell seeding onto their materials) and stimulated the cells electromechanically (0.05 Hz biphasic stimulation). Furthermore, they continued using this specialized differentiation medium with their materials, making it difficult to faithfully attribute such a cell maturation to the materials or the external cell stimulation. Au contraire, we can see a partial differentiation of H9c2 cells by solely using BC-Ppy composites as cellular substrates, undoubtedly highlighting the beneficial effect of these conductive biocomposites on cardiac maturation.

## Conclusions

Cardiac tissue engineering quests for biocompatible, mechanically robust, and flexible scaffolds that allow cell attachment and proliferation with adequate conductivity for the transmission of electrical signals to the entire myocardium. In this study, we created electroconductive nanofibrous scaffolds by incorporating Ppy NPs through *in situ* synthesis into the 3D interconnected bacterial cellulose fiber network. We investigated their use as cardiac tissue scaffolds. Polypyrrole NPs efficiently impart conductive properties to BC, increase surface roughness and thickness, and decrease transparency. BC-Ppy composites maintained flexibility up to 10 mM concentration and the 3D mesh-like structure in the entire range of concentrations.

The excellent cytocompatibility of BC-Ppy composites was confirmed with both primary human cardiac fibroblasts and H9c2 cardiomyoblasts. We demonstrated that the addition of polypyrrole improved the biocompatibility of BC materials since cardiomyoblasts showed enhanced cell viability, adhesion, morphology, and proliferation. H9c2 cells seeded on BC-Ppy showed an increased degree of differentiation to cardiomyocytes when combined with current chemical differentiation protocols, suggesting a synergistic effect of Ppy with the differentiation medium. Moreover, our results indicate that BC-Ppy materials alone (i.e., without chemical induction of maturation) may drive partial H9c2 differentiation, as evidenced by an enhanced expression of cardiac-specific proteins.

This work suggests that BC-Ppy nanofibrous scaffolds hold exceptional potential to be used as an *in vitro* platform for the differentiation of immature cardiac cells *in vitro* and encourages further investigations of the use of electroconductive BC-Ppy substrates in cardiac tissue engineering.

## Supporting Information Available

Schematic representations of experimental setup of mechanical testing and methodology of BC and BC-Ppy synthesis; further characterization of BC-Ppy materials (size distribution of BC nanofibers and Ppy NPs and stability of BC-Ppy); cytotoxicity of materials in “wet” format (viability and immunolabeled images); biocompatibility of BC-Ppy scaffolds with cardiac fibroblasts; toxicity of BC and BC-Ppy byproducts; comparison of H9c2 attachment on BC-Ppy nanocomposites and control culture plates; successful H9c2 maturation *in vitro*; expression of αMHC after differentiation treatment in control substrates (PDF)
