# Supplementary material for: Conductive Bacterial Nanocellulose-Polypyrrole Patches Promote Cardiomyocyte Differentiation
Source: ACS Appl Bio Mater. 2023 Jun 21;6(7):2860–74. doi: 10.1021/acsabm.3c00303 (PMC10354801; doi:10.1021/acsabm.3c00303)
Supplement: Supplementary file 1 — mt3c00303_si_001.pdf [file mt3c00303_si_001.pdf]

Supporting Information

Conductive Bacterial Nanocellulose-Polypyrrole patches promote cardiomyocyte differentiation

Sumithra Yasaswini Srinivasan ± [1], Marina Cler ±[2,3,4,5], Osnat Zapata-Arteaga [1], Bernhard Dörling, Mariano Campoy-Quiles [1], Elena Martínez [3,4,6], Elisabeth Engel [2,4,5], Soledad Pérez-Amodio *[2,4,5], Anna Laromaine *[1]

*Email: sperez@ibecbarcelona.eu
*Email: alaromaine@icmab.es

[1] *Institute of Material Science of Barcelona (ICMAB), CSIC, Campus UAB, 08193 Bellaterra, Spain*

[2] *IMEM-BRT group, Departament de Ciència i Enginyeria de Materials, Universitat Politecnica de Catalunya, 08028 Barcelona, Spain*

[3] *Biomimetic Systems for Cell Engineering, Institute for Bioengineering of Catalonia (IBEC), The Barcelona Institute of Science and Technology, 08028 Barcelona, Spain*

[4] *CIBER en Bioingenieria, Biomateriales y Nanomedicina, CIBER-BBN, 28029 Madrid, Spain*

[5] *Biomaterials for Regenerative Therapies, Institute of Bioengineering Catalunya (IBEC), The Barcelona Institute of Science and Technology, 08028 Barcelona, Spain*

[6] *Department of Electronics and Biomedical Engineering, University of Barcelona (UB), 08028 Barcelona, Spain*

± Those authors contributed equally to the work *corresponding authors

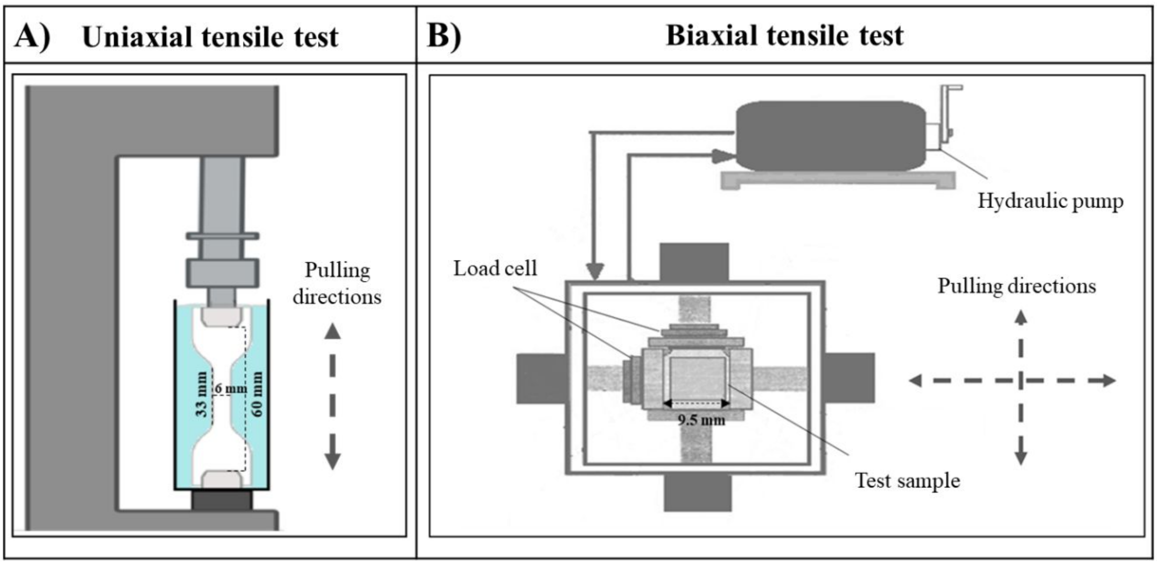

Figure S1 – Experimental setup for the tensile testing. A) Mechanical testing of BC and BC-Ppy materials in uniaxial and B) biaxial directions.

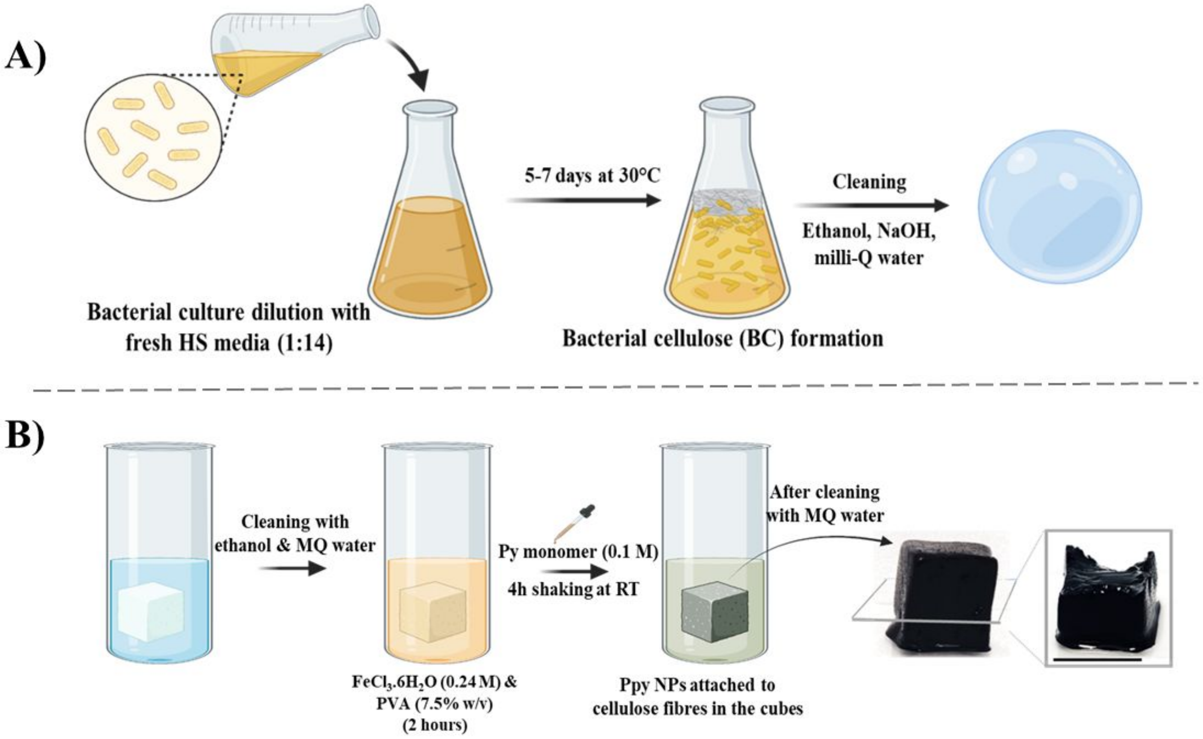

Figure S2 – Schematic representation of experimental procedures. A) Bacterial nanocellulose production using *K. xylinus* bacterial cultures in the laboratory. B) Synthesis of BC-Ppy from commercial nanocellulose cubes

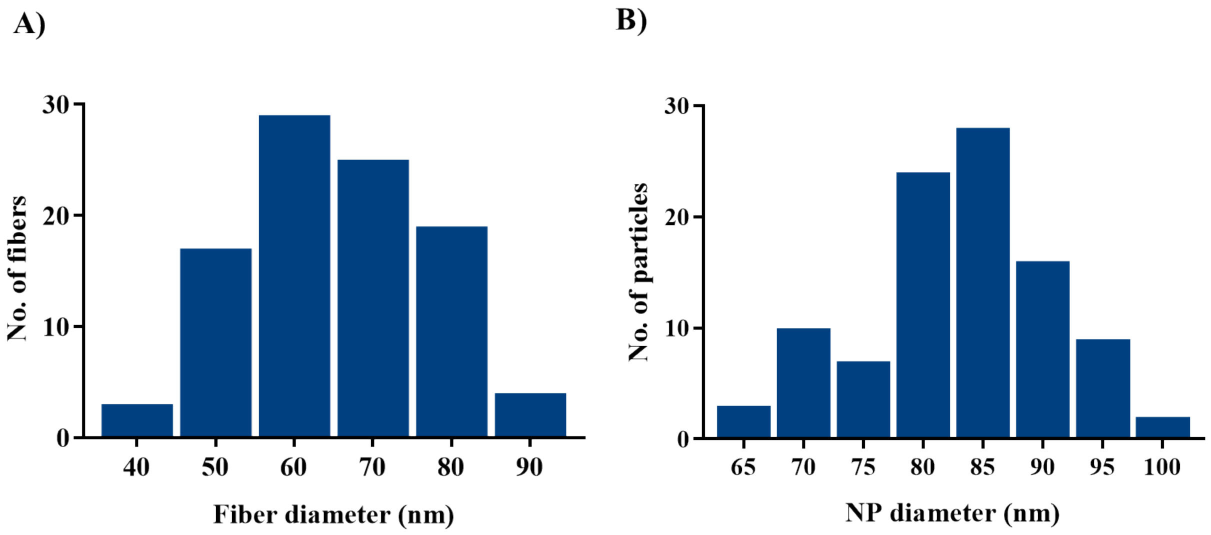

Figure S3 – Size characterization of BC-Ppy components. A) Size distribution of BNC fibers (N=97). B) Ppy NPs (N=99) plotted from TEM images.

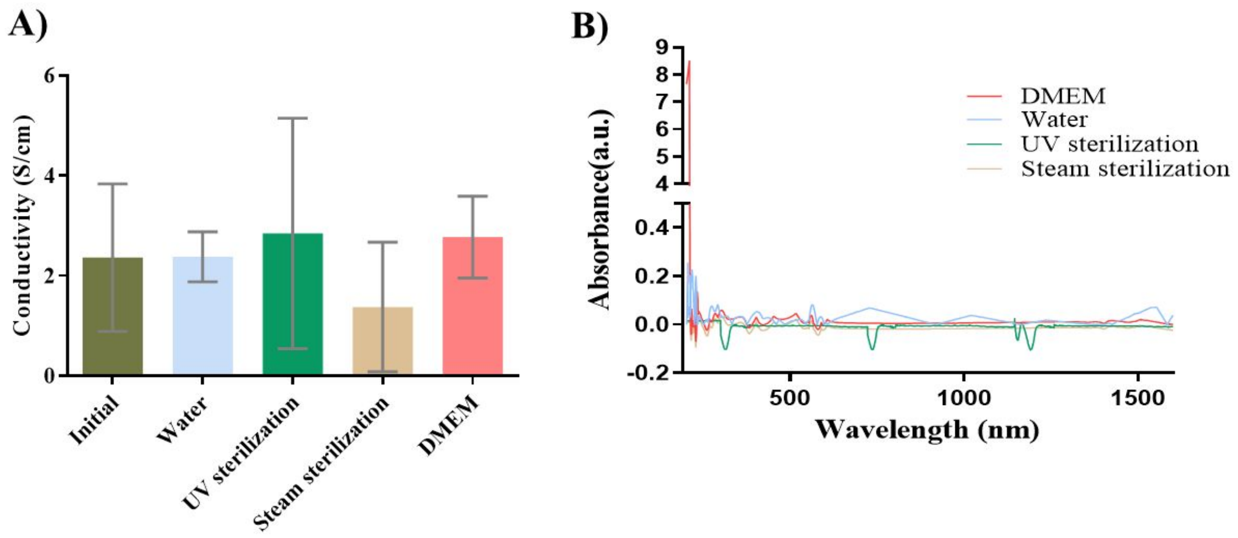

Figure S4 – Stability of BC-Ppy in different experimental conditions. A) Conductivity of the scaffolds after UV sterilization, autoclaving (steam sterilization), DMEM, or water incubation. B) UV-Vis-NIR absorption spectra after the same conditions.

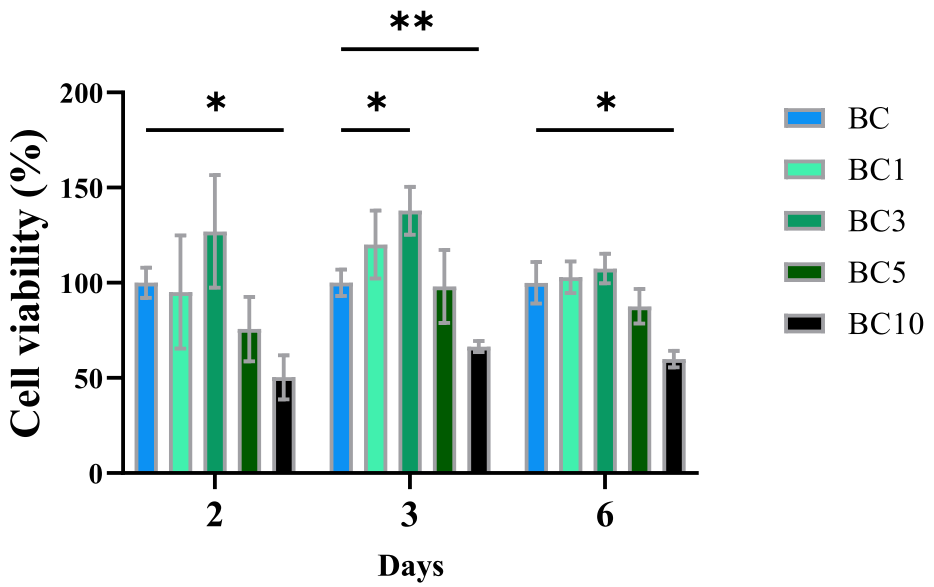

Figure S5 – Cytotoxicity of H9c2 on BC-Ppy scaffolds. Cell viability of cardiomyoblasts during 2,3, and 6 days of culture onto "wet" scaffolds analyzed by MTT (n=3 at day 2 and n=4 at days 3 and 6; data normalized to the absorbance values obtained in plain BC materials at each timepoint).

<u>**Supplementary information: Figure S6 – Cardiac fibroblasts viability and attachment to BC-Ppy scaffolds.**</u>

Human adult cardiac fibroblasts (HCFs) were seeded on BC and BP2 (2 mM Pyrrole) and assessed by LIFE/DEAD assay staining. Viability of HCFs after 24 hours of culture was high (≈95%) and similar in BC and BC-Ppy composites (Figure S6A, S6B). Unlike H9c2 (Figure 6), HCFs properly attach to BC materials as evidenced by a similar cell density regardless of the

presence or not of Ppy (Figure S6C). Culturing HCFs for longer periods (72h) rendered equivalent results and the characteristic spindle-like morphology of HCFs was retained in both BC and BC-Ppy biomaterials (Figure S6D).

Following our results with myoblasts, we also tested whether high concentrations of Ppy could affect HCFs behavior and attachment to the BC-Ppy materials. For this, we cultured HCFs onto BP10 scaffolds, which caused a decrease in H9c2 cell density and attachment given its high Ppy concentration (Figure 6). To track alive cells over time, we labelled HCFs with Vybrant™ DiO Cell-Labeling Solution (V22886, Invitrogen™). HCFs properly attached to BP10 materials and depicted similar cell number and morphology as cells grown onto plain BC substrates (Figure S6E). Cardiac fibroblasts perfectly attach and grow on plain BC scaffolds and can withstand higher concentrations of conductive Ppy than H9c2, indicative of a less sensitive behavior in comparison with cardiomyoblasts.

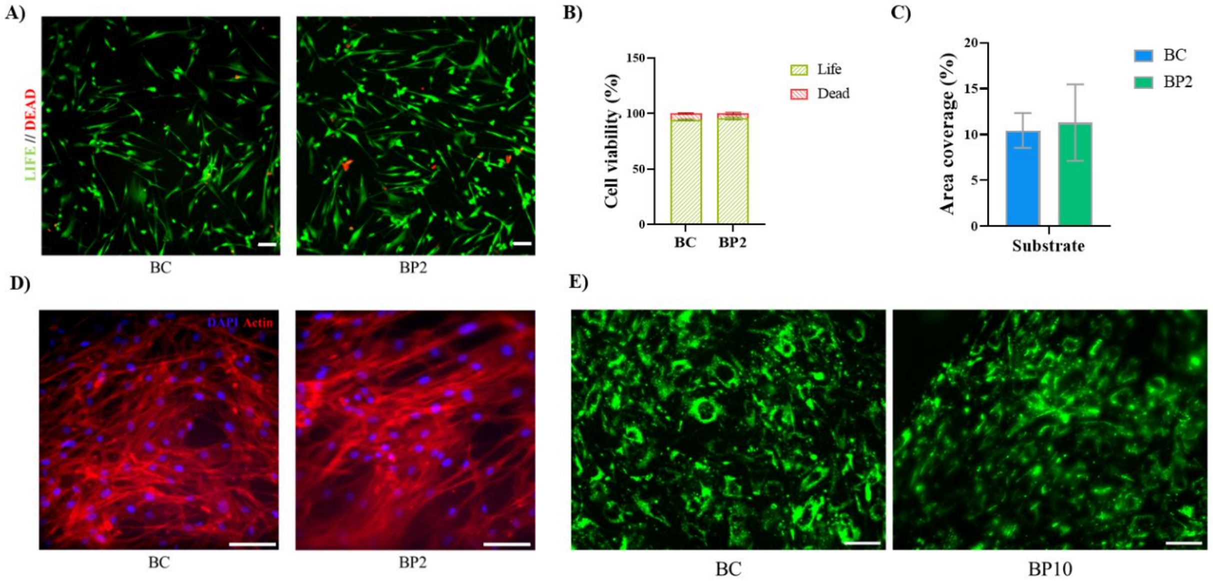

Figure S6 – Cardiac fibroblasts viability and attachment to BC-PPy scaffolds. A) LIFE/DEAD Staining of HCFs cultured 24 hours onto BC and BP2 scaffolds. B) Quantification of LIFE/DEAD images (n=3). C) Area coverage of cells adhered to BC and BP2 scaffolds after 24h of culture (n=3). D) Attachment of HCFs onto BC and BP2 scaffolds after 72h of culture (n=4, representative images shown). E) HCFs cultured onto BC and BP10 scaffolds for 48 hours (n=4, representative images shown). Scale bars: 100 µm.

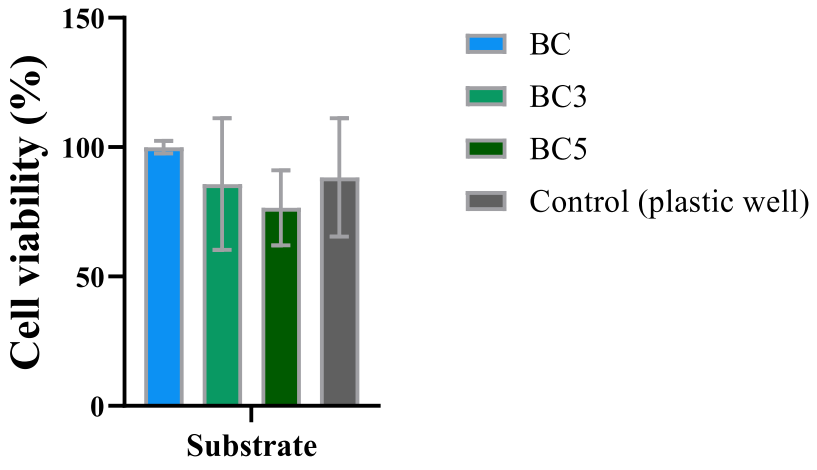

Figure S7 – Toxicity of BC and BC-Ppy by-products on cardiomyoblasts. Cell culture medium was incubated with the scaffolds and empty material-free wells were used as control. After an overnight incubation, media was collected and H9c2 cells were further incubated during 24h with the collected media before checking viability by MTT (n=4; data normalized to the viability values of H9c2 incubated with media from plain BC scaffolds).

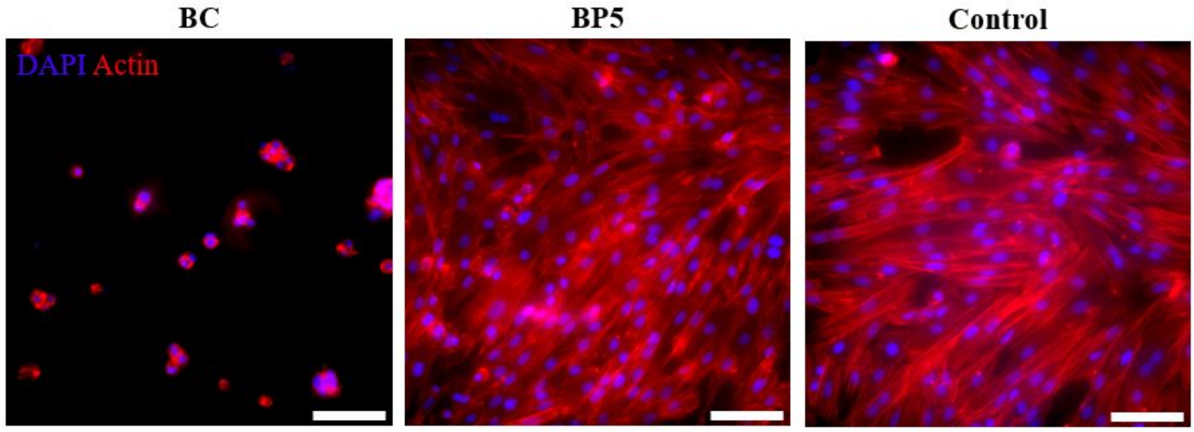

Figure S8 – Fluorescence microscopy images of H9c2 onto BC-Ppy composites. Cells grown onto BC-Ppy (BP5) composites retain the characteristic H9c2 spread-out morphology seen in conventional plastic culture plates (control). H9c2 cells in plain BC acquired an atypical, rounded morphology. Scale bars: 100 µm.

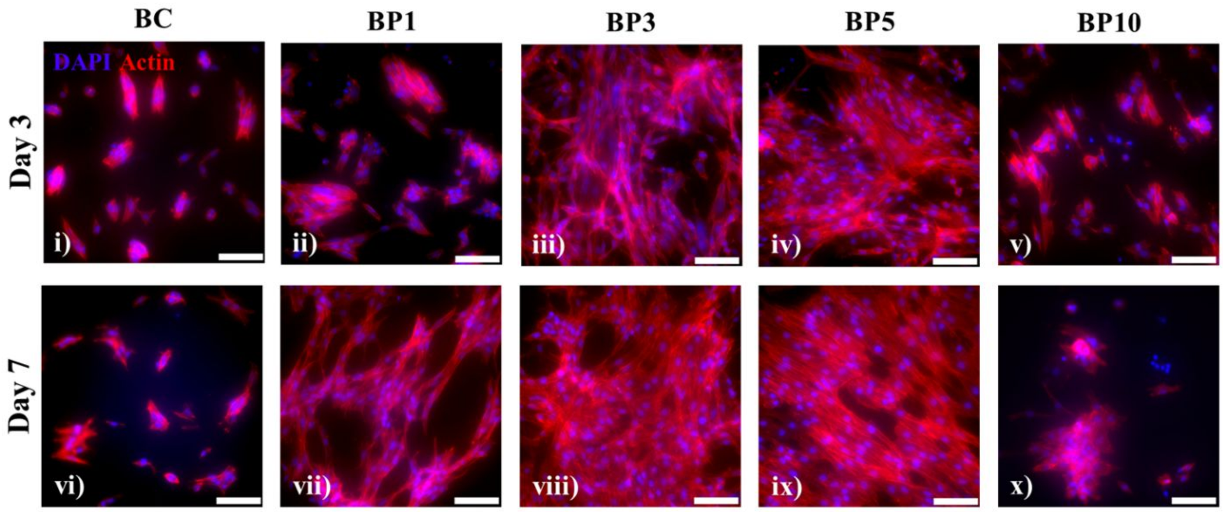

Figure S9 – H9c2 cells onto "wet" BC-Ppy scaffolds. Fluorescence microscopy images of the cell populations seeded on "wet" nanocellulose composites (without drying) after 3 and 7 days of culture. Scale bars: 100 µm.

Supplementary information: Figure S10 – Generating cardiomyocyte cell lineages derived from H9c2 cardiomyoblasts.

Two concentrations of RA were tested to induce differentiation of H9c2: 10 nM and 1 µM. After 6 days of treatment, the first indicatives of H9c2 differentiation could be clearly seen through phase-contrast microscopy. Incubation of H9c2 cells for 6 and 10 consecutive days with differentiation media resulted in cell fusion and development of larger cells, a morphological sign of H9c2 differentiation[41] (Supplementary Figure S10A). H9c2 proliferation decreased, a signature of mature cardiomyocytes[42] (Supplementary Figure S10B). H9c2 also increased their expression of the cardiac marker Cardiac Troponin T (cTnT) after 6 days of incubation with differentiation media, as checked by Western blotting (Supplementary Figure S10C). Increase of cTnT expression in differentiated H9c2 cells was further validated by immunostaining after 6 and 10 days of treatment (Supplementary Figure S10D, S10E). Similarly, immunofluorescence images revealed a decrease in the proliferative capacity of H9c2 cells after induction of differentiation, evidenced by the decrease of the proliferative marker Ki67 (Supplementary Figure S10D, S10F). All in all, here we provide solid evidence of the successful differentiation of H9c2 to cardiomyocyte-like cells by using two concentrations of RA (10 nM and 1 µM) in the differentiation media and using two treatment durations (6 and 10 days).

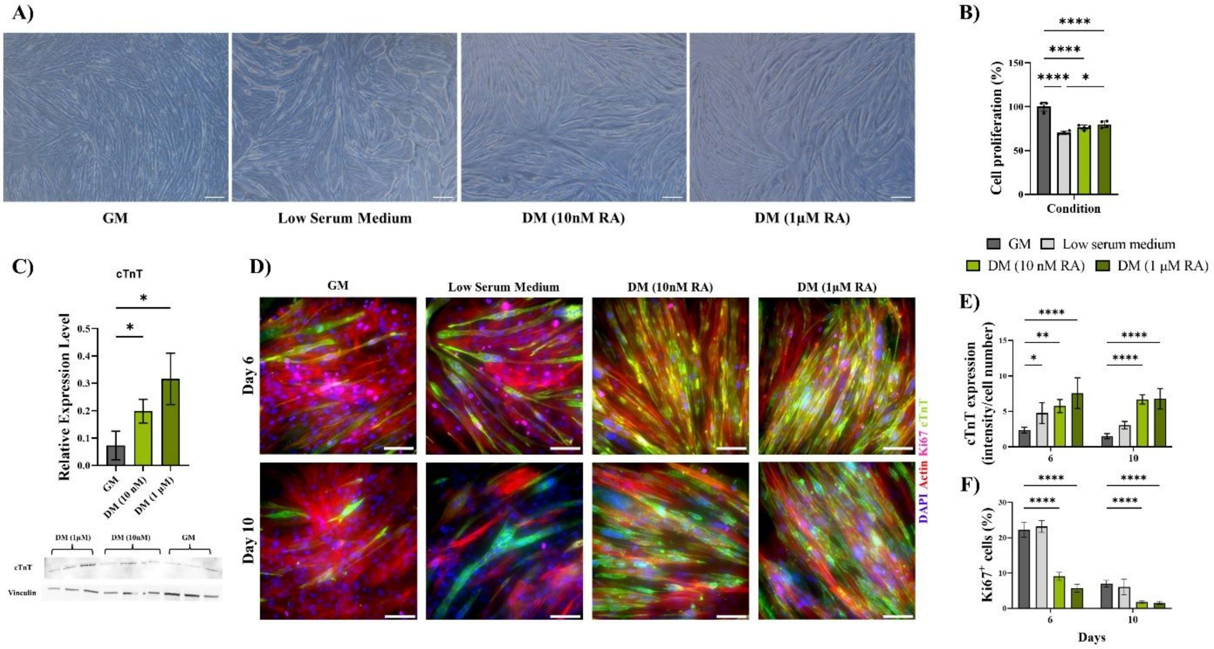

Figure S10 – Generating cardiomyocyte cell lineages from H9c2 cardiomyoblasts. A) Representative images of H9c2 cells after 6 days of incubation with growth medium, low serum medium (1% FBS), and differentiation medium (low serum medium supplemented with RA). B) H9c2 proliferation after 6 days of incubation with different media (n=4). C) cTnT expression after 6 consecutive days of treatment with two differentiation media (containing 10 nM and 1 µM RA) (n=3). D) Immunostaining images of H9c2 cells after 6 and 10 days of incubation with growth media, low serum media, and differentiation media. E) Quantification of cTnT expression after 6 and 10 days of treatment with different media (n=4). F) Percentage of Ki67^{+} H9c2 cells after 6 and 10 days of incubation with different media (n=4). GM= Growth Medium; DM= Differentiation Medium. Scale bars: 100 µm.

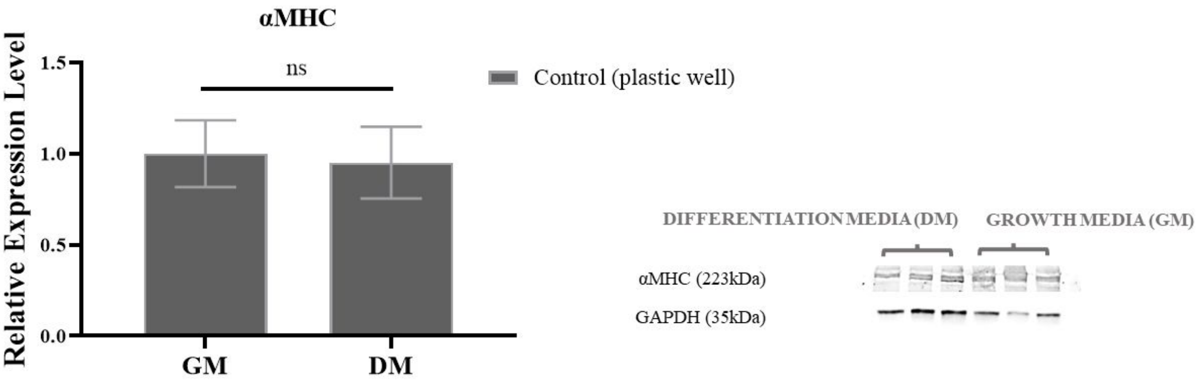

Figure S11 – αMHC expression in control culture plates. Protein expression of αMHC of H9c2 cells cultured in normal growth media and differentiation media for 6 consecutive days in control substrates (material-free plastic wells). GAPDH was used as the loading control. (n=3, protein expression data normalized to intensity values of cells grown with growth medium). GM= Growth Medium; DM= Differentiation Medium.
